# An *Alfin-like* gene from *Atriplex hortensis* enhances salt and drought tolerance and abscisic acid response in transgenic Arabidopsis

**DOI:** 10.1038/s41598-018-21148-9

**Published:** 2018-02-09

**Authors:** Jian-Jun Tao, Wei Wei, Wen-Jia Pan, Long Lu, Qing-Tian Li, Jin-Biao Ma, Wan-Ke Zhang, Biao Ma, Shou-Yi Chen, Jin-Song Zhang

**Affiliations:** 1 State Key Lab of Plant Genomics, Institute of Genetics and Developmental Biology, Chinese Academy of Sciences, Beijing, 100101 China; 2 Key Laboratory of Biogeography and Bioresource in Arid Land, Xinjiang Institute of Ecology and Geography, Chinese Academy of Sciences, Urumqi, Xinjiang China; 3 University of Chinese Academy of Sciences, Beijing, 100049 China

## Abstract

*Alfin-like* (*AL*) is a small plant-specific gene family with prominent roles in root growth and abiotic stress response. Here, we aimed to identify novel stress tolerance *AL* genes from the stress-tolerant species *Atriplex hortensis*. Totally, we isolated four *AhAL* genes, all encoding nuclear-localized proteins with cis-element-binding and transrepression activities. Constitutive expression of *AhAL1* in Arabidopsis facilitated plants to survive under saline condition, while expressing anyone of the other three *AhAL* genes led to salt-hypersensitive response, indicating functional divergence of *AhAL* family. *AhAL1* also conferred enhanced drought tolerance, as judged from enhanced survival, improved growth, decreased malonaldehyde (MDA) content and reduced water loss in *AhAL1*-expressing plants compared to WT. In addition, abscisic acid (ABA)-mediated stomatal closure and inhibition of seed germination and primary root elongation were enhanced in *AhAL1*-transgenic plants. Further analysis demonstrated that AhAL1 could bind to promoter regions of *GRF7*, *DREB1C* and several group-A *PP2C* genes and repress their expression. Correspondingly, the expression levels of positive stress regulator genes *DREB1A*, *DREB2A* and three *ABFs* were all increased in *AhAL1*-expressing plants. Based on these results, *AhAL1* was identified as a novel candidate gene for improving abiotic stress tolerance of crop plants.

## Introduction

Plant adaptation to long-term environmental stress requires integrated changes in regulation of functional genes for physiological adjustments in both cellular and organismal level. These changes contribute to plants for coordination of normal growth and stress response. Using microarray and transcriptome sequencing analysis, varieties of genes have been found to be induced or reduced during encounters of harsh conditions (drought, salinity, cold, heat and etc)^1–4^. Usually, transcription factors play key roles in the regulatory networks underlying plant responses to these stresses. Until now, numerous stress-related transcription factors have been characterized in plants, including dehydration-responsive element-binding (DREB), NAC, MYB/MYC, WRKY, AP2/ERF, bZIP, Alfin-like (AL), and some other families^5–12^. Among them, the AL family belongs to a plant-specific subfamily of plant homeodomain (PHD) finger proteins and was first identified as a kind of transcription factor family in alfalfa (*Medicago sativa* L.)^13^. Except for plants, none of AL proteins were ever reported in animals, fungi, and prokaryotes.

All AL proteins contain three domains: the N-terminal conserved N domain (DUF3594), the C-terminal conserved PHD-finger domain (Cys4HisCys3-type) and the V domain (a variable region between the two conserved domains)^14^. The PHD finger was first discovered in the Arabidopsis (*Arabidopsis thaliana*) protein HAT3.1 (Histone acetyltransferases 3.1)^15^. With high similarity to the Cys3HisCys4-type RING finger, the PHD finger (Cys4HisCys3-type) can also bind two zinc atoms and has similar solution structure^16^. This feature enables the PHD finger to bind some nuclear protein partners^17–19^. In fact, Arabidopsis ING and Alfin-like proteins can bind to the active histone markers (H3K4me3/2) via PHD fingers, and thus may participate in chromatin regulation^20^. Besides its roles in mediating protein interactions, PHD domain seems to function in nuclear localization of AL proteins^21^. In soybean, the Alfin-like protein GmPHD6 interacted with itself and the other four GmPHDs except for GmPHD2 in yeast cells, possibly through N domain and V domain rather than the PHD finger domain^21^. In addition to binding proteins, most AL proteins can also bind to the conserved cis-element GNGGTG/GTGGNG through N domain^13,14,21^. Exceptionally, the Arabidopsis AL6 is unable to bind the conserved cis-element. Further analysis revealed that the loss of DNA-binding ability arises from variations of two amino acids at positions 34 and 35 in N domain^14^.

Since the first isolation of Alfin1 cDNA from salt-tolerant alfalfa cells^22^, studies were mainly focused on exploring the roles of AL family in plant response to salt stress. As a salt-responsive candidate gene, *Alfin1* was isolated from the salt-tolerant alfalfa cells. Alfin1 bound efficiently to three regions in the promoter of salt-inducible gene *MsPRP2*, each containing the conserved element GNGGTG/GTGGNG^13^. Transgenic analysis in alfalfa demonstrated that overexpression of *Alfin1* increased *MsPRP2* mRNA levels in roots and enhanced salt tolerance of the transgenic plants^23–25^. The expression of most *ALs* genes from soybean and Arabidopsis are stress-responsive, with some differences in stimuli type and expression level^14,21^. Constitutively expressing *GmPHD2*, an *Alfin-like* gene from soybean, promoted the salt tolerance of transgenic Arabidopsis plants, probably by eliminating the excessive stress-induced Reactive Oxygen Species (ROS)^21^. Another Alfin-like protein GmPHD5 from soybean seems to be an important regulator of the histone methylation-acetylation crosstalk in response to salt stress. Under salinity condition, GmPHD5 is likely to recruit chromatin remodeling factors and transcription factors to regulate the expression of stress-inducible genes such as *GmRD22* and *GmGST*^26^. AL5 from Arabidopsis could bind directly to promoters of various downstream genes and inhibit expression of these negative regulators for plant adaptation to salt, drought and freezing stress^14^. These findings suggest that AL family proteins play important roles in regulating plant responses to environmental stimuli.

Molecular evolution analysis of AL proteins in two Arabidopsis species and a salt-tolerant relative *Thellungiella halophila* found that natural selection has occurred during the evolution of *ALs* genes^27^. We propose that natural selection of *ALs* genes might contribute to the high salt-tolerance abilities of *T. halophila* and some other halophyte flora. *Atriplex hortensis* (*A. hortensis*), also called mountain spinach, can tolerate harsh environments including drought, cold and high salinity. Previously, we have isolated functional genes from this halophyte species and these genes conferred improved salt/drought tolerance in transgenic model and crop plants^28–31^. A DREB-like transcription factor gene was also isolated from *A. hortensis* and could promote stress tolerance when expressed in tobacco plants^32^. In recent years, we have analyzed functions of *AL/PHD* family genes from Arabidopsis and soybean in stress response^14,21^. In this study, we further isolated four *AL* genes from *A. hortensis* and investigated their functions in plant response to abiotic stresses. The four AhALs are nuclear-localized, could bind to the cis-element “GTGGAG”, and show transcriptional repression activity. Constitutive expression of *AhAL1* improved plants tolerances to salt and drought stress, and also enhanced abscisic acid (ABA) responses. In contrast, the other three *AhALs* all conferred hypersensitive response to salt stress. Our study reveals functional divergence of different AL members in abiotic stress response and identified *AhAL1* as a potential candidate gene for enhancing stress tolerance of crop plants.

## Results

### Identification of *Alfin-like* genes from *A. hortensis*

**Figure 1:**
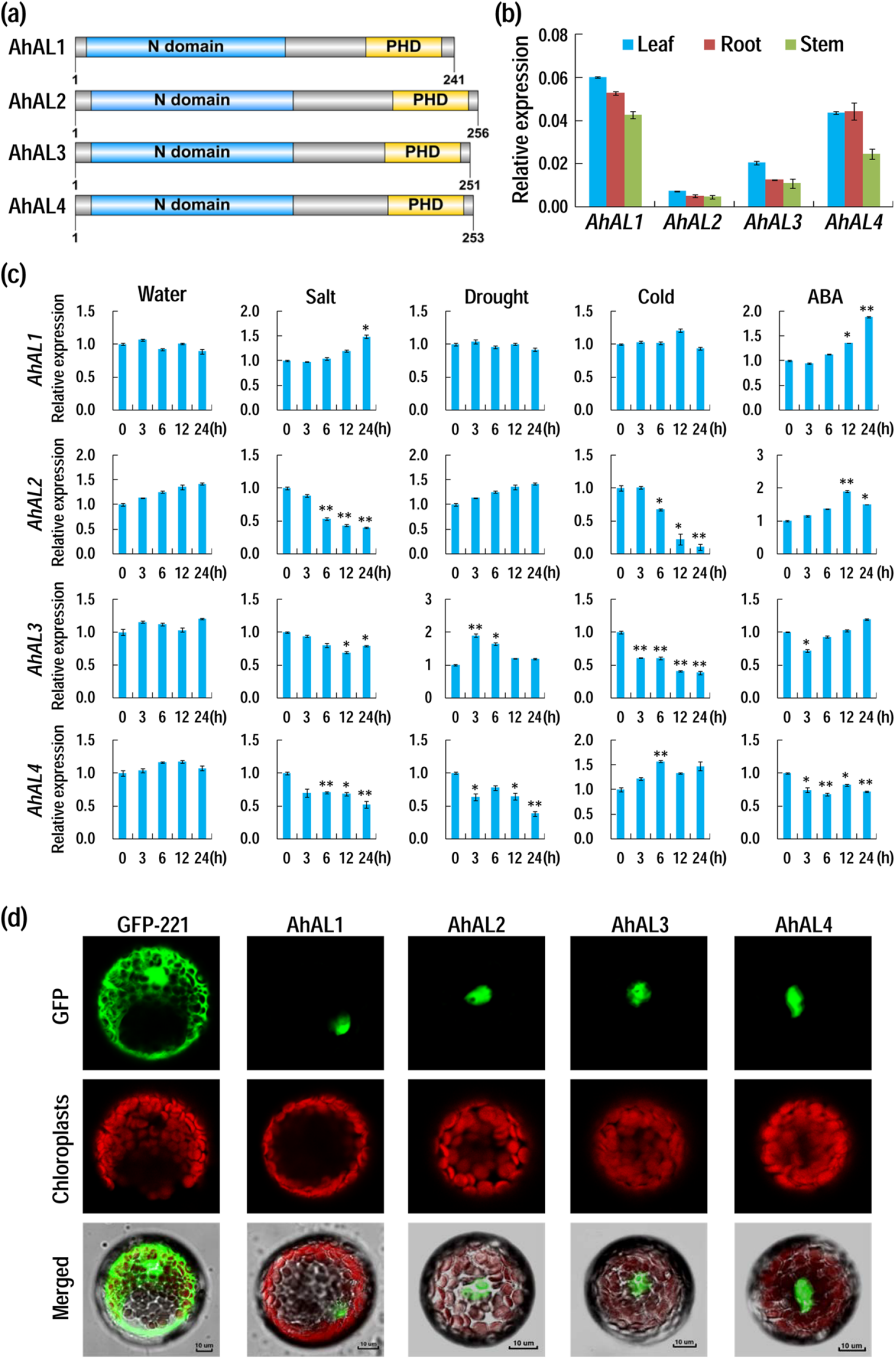
Protein structure, expression pattern and subcellular localization of AhALs. (**a**) Protein structure of AL proteins from *A. hortensis*. Bars in blue color indicate the conserved N domain (DUF3594), and bars in yellow color indicate the PHD-finger domain. A variable V region is located between the two conserved domains. (**b**) Expression of *AhALs* in leaf, root and stem. (**c**) Expression patterns of *AhALs* under various stress treatments. Two-week-old *A. hortensis* seedlings were treated with 200 mM NaCl, drought, cold (0 °C) and 100 μM ABA, and total RNA was extracted for qRT-PCR analysis. Treatment in water without any stress was set as a control. Values are relative to the expression level at 0 h. For qRT-PCR detection in (**b**) and (**c**), *A. hortensis actin7* gene *AhACT7* was used as the endogenous control. Values are means ± SD (n = 3). Student’s *t* tests between 0 h and each time point (**c**) were performed (*P < 0.05; **P < 0.01). Three biological replicates were performed with similar results, and one of them was shown. (**d**) Subcellular localization of AhALs revealed by fluorescently confocal imaging of AhAL-GFP fusion proteins or GFP control in Arabidopsis protoplasts (Bar = 10 μm).

Four full-length *Alfin-*like genes were isolated from halophyte *A. hortensis* using degenerate RT-PCR and RACE methods. These genes encoded Alfin-like proteins and named as *AhAL1* to *AhAL4*. Similar to the ALs from Arabidopsis and GmPHDs from soybean^14,21^, all the four AhALs contain a conserved N domain at the N-terminus, a PHD finger domain with conserved C4HC3 residues at the C-terminus and a variable V region between the two conserved domains (Fig. 1a, Supplementary Fig. S1a). Sequence alignment revealed that AhAL proteins had 59% to 77% identities with each other (Supplementary Fig. S1b). Phylogenetic analysis of the AhALs with ALs/PHDs from other plant species indicated that each AhAL was clustered into different groups and AhAL1 was more distantly related to the other members of the AhAL family (Supplementary Fig. S2).

### Expression pattern and subcellular localization of AhALs

Expression of *AhALs* in different organs of *A. hortensis* was examined (Fig. 1b). All the four *AhAL* genes had slightly higher expression levels in leaf and root compared to those in stem. Among the four *AhAL* genes, *AhAL1* and *AhAL4* are more highly expressed than the *AhAL2* and *AhAL3*, and the *AhAL2* is the lowest expressing gene.

We then tested whether the four *AhAL* genes from the halophyte *A. hortensis* are responsive to different stresses using quantitative RT-PCR (Fig. 1c). The transcript of *AhAL1* was slowly but constantly increased within 24 h under salt and ABA treatments, but almost unchanged under drought and cold treatments. The expression of *AhAL2* was moderately induced by ABA treatment, but dramatically reduced by salt and cold treatments. Under drought treatment, the transcript of *AhAL3* was apparently increased within 3 h treatment and then decreased gradually. In contrast, the expression of *AhAL3* was inhibited slightly by salt and ABA treatments and significantly by cold treatment. The *AhAL4* was slightly upregulated by cold stress but distinctly downregulated by salt, drought and ABA treatments. These results showed that the four *AhAL* genes exhibited diverse expression patterns under different stress treatments, indicating each of them may have specific roles in plant stress response.

Subcellular localization of four AhAL proteins was examined by fusing with GFP and transiently expressed in Arabidopsis protoplasts. The green fluorescence signal of AhAL-GFP proteins were detected only in the nucleus under laser confocal microscope (Fig. 1d), indicating that AhALs are nuclear-localized proteins and possibly function as transcription regulators like the ALs from Arabidopsis and soybean^14,21^.

### Transcriptional regulatory activity and DNA binding ability of AhALs

**Figure 2:**
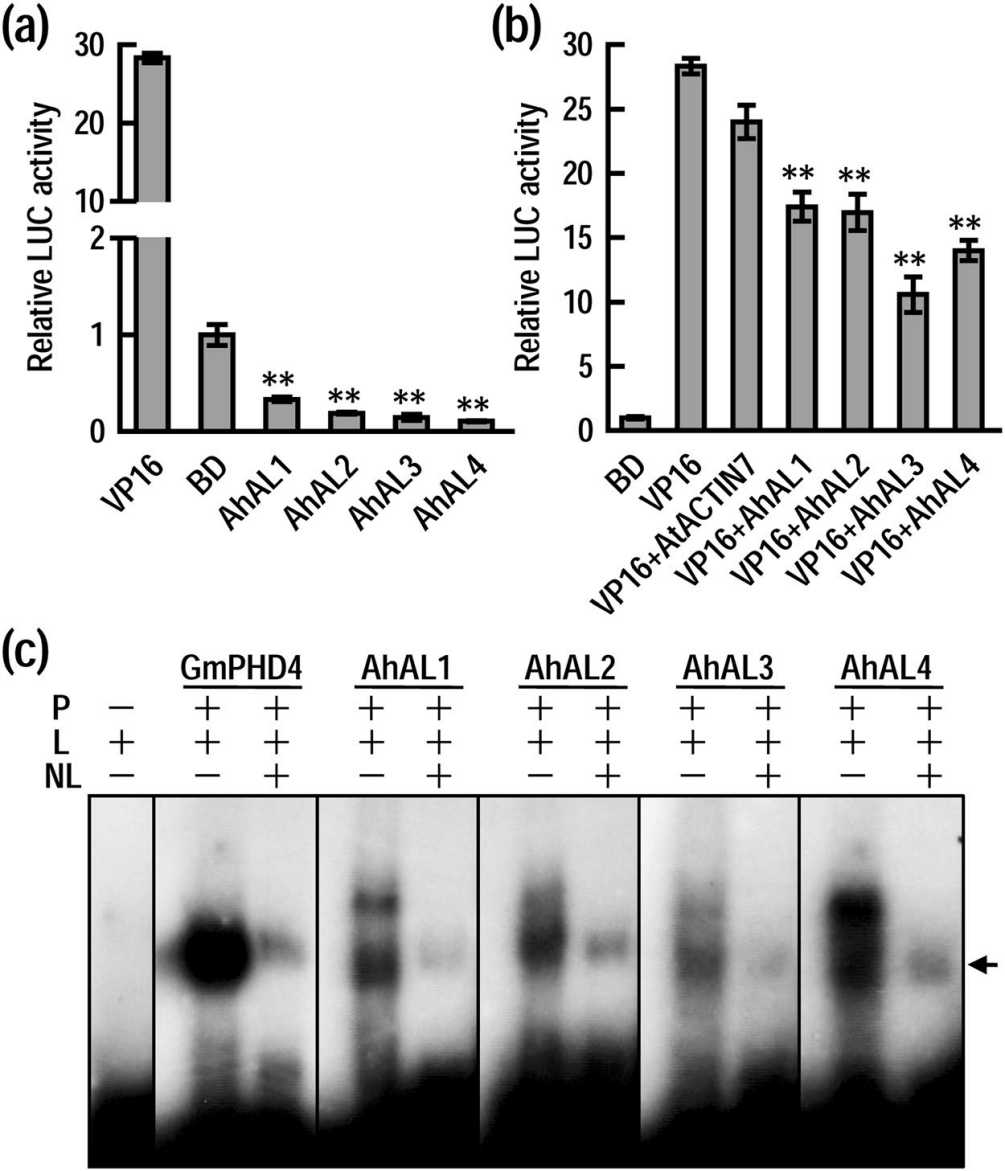
Assays of transcriptional regulation and cis-element binding activities of AhAL proteins. (**a**) Effects of AhALs on the reporter gene expression as revealed by relative LUC activities in Arabidopsis protoplasts. BD (GAL4 DNA-binding domain)-VP16 and BD alone were used as positive and negative control respectively. Plasmids of effectors (35S-BD-AhALs), reporter (5 × GAL4-LUC) and internal control (pPTRL) were used with a ratio of 6:6:1. Data shown are relative values compared to the negative control BD. Student’s *t* tests between BD and AhALs were performed (**P < 0.01). (**b**) Effects of AhALs on VP16-mediated transactivation of *LUC* gene. AtACTIN7 was used as a null control. Plasmids of effectors (35S-BD-VP16 + 35S-AhALs), reporter (5 × GAL4-LUC) and internal control (pPTRL) were used with a ratio of (6 + 6):6:1. Data shown are relative values compared to the negative control BD. Student’s *t* tests between VP16 and VP16 + AhALs were performed (**P < 0.01). (**c**) Cis element “GTGGAG”-binding abilities of AhALs as revealed by gel shift assay. GmPHD4 was used as a positive control. P, proteins; L, radiolabeled probe containing 2 × GTGGAG (AACTTCATCTAGGTGGAGATGTATGATGTACTACTACATGTATCGTGGAGTACAACATC); NL, unlabeled probe (added in ten-fold of the labeled probes). Arrows indicate bands of protein/DNA complexes. Blots cropped from different parts of the same exposure, or from different exposures were made explicit using black dividing lines.

In our previous studies, most AL/PHD family members from soybean and Arabidopsis were identified as transcriptional repressors to restrain gene expression^14,21^. Here we used a dual-luciferase reporter assay system to determine the transcriptional regulation activities of AhALs in Arabidopsis protoplasts (Fig. 2a). In comparison with the positive control of transcriptional activator VP16, four AhALs showed no transactivation activity. Conversely, these AhALs all exhibited repression activities compared to the BD control. To further confirm the transcriptional repression activities of AhALs, we examined whether AhALs could suppress the transactivation activity of VP16 when they were co-expressed in Arabidopsis protoplasts. As seen in Fig. 2b, the VP16-driven luciferase activity was repressed significantly by each of AhALs, while AtACTIN7 showed no significant effect on the activity of VP16. These results indicate that AhALs may function as transcriptional repressors to regulate gene expression.

AL/PHD family proteins from alfalfa, soybean and Arabidopsis could bind to the core cis-element GNGGTG/GTGGNG and thus to regulate gene transcription^13,14,21^. Gel-shift assay was performed to examine whether AhALs could also bind to this core element. GST-tagged AhALs fusion proteins were expressed in *E.coli* (BL21) and purified (Supplementary Fig. S3). DNA fragments containing two copies of GTGGAG were annealed, end-labeled with [γ-^32^P]-dATP and used as DNA binding probe for gel-shift assay (Fig. 2c). As seen in Fig. 2c, all AhAL proteins formed distinctive complexes with the radiolabeled probes, and this binding could be largely removed by addition of excess unlabeled DNA probes. These results indicate that all the four AhALs can specifically bind to the core cis-element GTGGAG.

### AhAL1 confers enhanced salt and drought tolerance in transgenic plants

Since the *AhALs* genes are responsive to stresses and/or ABA treatment, a series of *AhAL*-transgenic Arabidopsis lines were generated and their performance under different stress treatments were examined.

**Figure 3:**
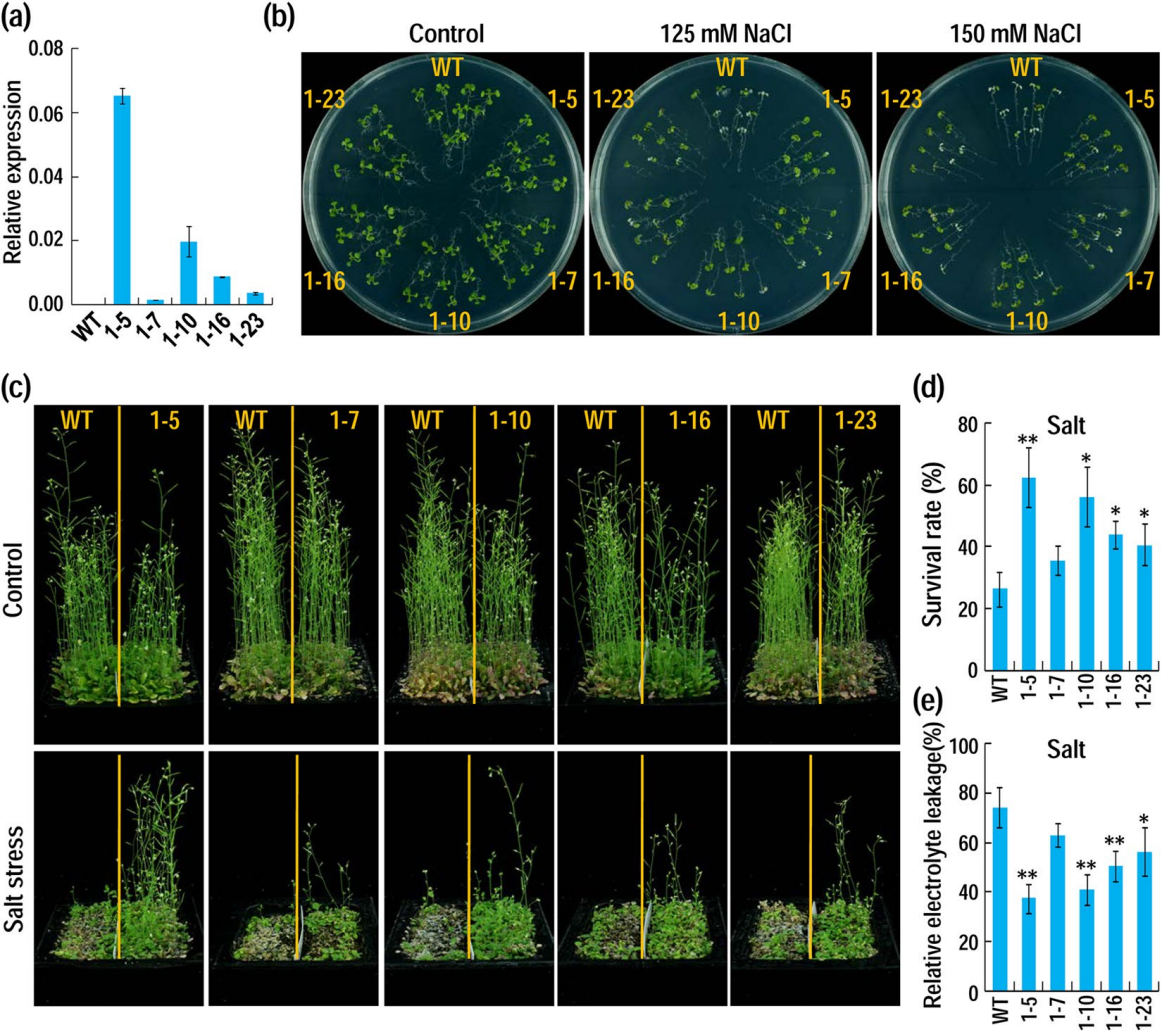
Constitutive expression of *AhAL1* enhances plants tolerance to salt stress. (**a**) Transcripts level of *AhAL1* in WT (Col-0) and *AhAL1* transgenic lines. Arabidopsis *actin 2* gene *AtACT2* was used as the endogenous control. Values are means ± SD (n = 3). (**b**) Plant phenotypes after salt treatment on 1/2 MS. Five-day-old seedlings of different *AhAL1*-transgenic lines and WT were transplanted to NaCl-containing 1/2 MS for stress treatment. When WT seedling appeared obviously stressed phenotype (about one week), photographs are taken. (**c**) Performance of plants after recovery from soil-salinity stress. Twelve-day-old seedlings growing in soil were watered with 250 mM NaCl solution to soil capacity (For each 10 × 10 cm pot, left half was WT, while right half was *AhAL1*-transgenic line; all transgenic lines accompanied with WT were placed in one tray for watering and salt-treatment). After treatment for two weeks, salinity soil was dialyzed against water to remove most of salts for growth recovery. After recovery for 10 d, photographs were taken and survival rates were calculated. (**d**) Survival rate of plants after recovery from soil-salinity stress. Values are means ± SD from three independent experimental groups (n = 100). (**e**) Relative electrolyte leakage in plants after salt treatment with 150 mM NaCl. Values are means ± SD (n = 3). For all data, asterisks indicate significant differences from the WT (Student’s *t* test; *P < 0.05; **P < 0.01).

Five *AhAL1*-transgenic lines were selected for examining the roles of *AhAL1* in plant adaptation to salt and drought stresses (Fig. 3a). Transplanted to 1/2 MS plates containing 125 or 150 mM NaCl for 7 d, all *AhAL1*-transgenic plants exhibited obviously enhanced salt tolerance, as judged from more survived seedlings than the wild type (WT) (Fig. 3b). Salt-tolerance abilities of these plants in soil salinity condition were also measured. After watering with 250 mM NaCl solution for two weeks and recovery for 10 d, the majority of WT plants were dead, whereas *AhAL1*-transgenic plants were survived much better (Fig. 3c and d). The electrolyte leakage was also measured and the level of this parameter is apparently lower in the stressed-transgenic lines than that in the stressed-WT, reflecting less cell membrane damage in *AhAL1*-transgenic plants after salt stress (Fig. 3e). Generally, salt-tolerance ability was coincident with the expression level of *AhAL1*. Two *AhAL1*-high-expressing lines 1–5 and 1–10 were most salt-tolerant, while the lowest expressing line 1–7 showed no significant promotion of salt tolerance. In addition, seed germination rates were slightly but significantly higher in the *AhAL1*-transgenic plants than that in WT (Supplementary Fig. S4). These data indicate that constitutive expression of *AhAL1* improved salt tolerance in transgenic Arabidopsis plants. Unlike *AhAL1*, the other three *A. hortensis AL* genes *AhAL2*, *AhAL3* and *AhAL4* all conferred hypersensitivity to salt stress when constitutively expressed in Arabidopsis, as revealed by decreased germination rate, fresh weight and survival rate, and more severe epinasty phenotype in transgenic plants compared to WT under salt treatment (Supplementary Figs S5 to S10). These data indicate functional divergence of different AhAL member in plant response to salt stress.

**Figure 4:**
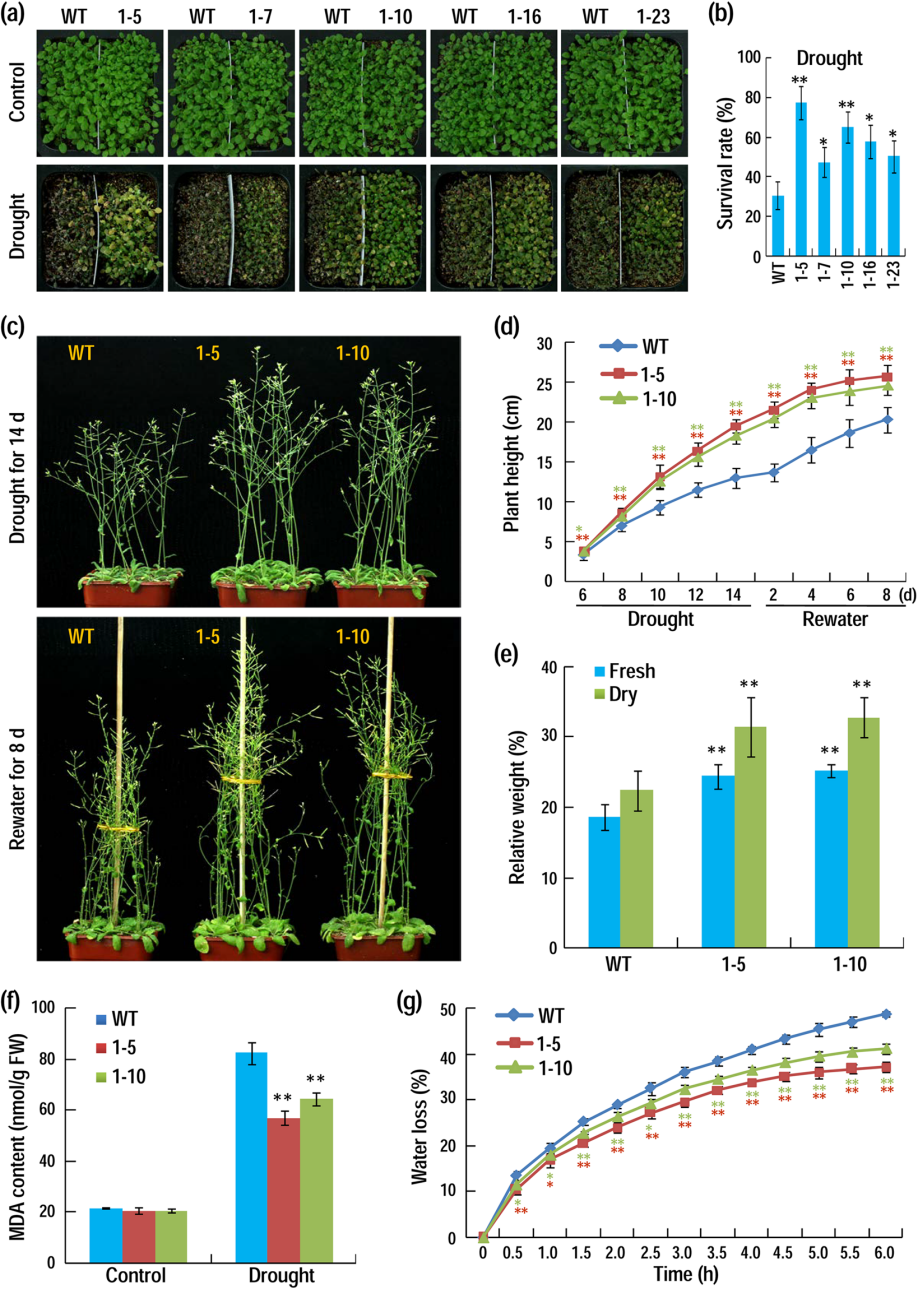
Constitutive expression of *AhAL1* promotes plants tolerance to drought stress. (**a**) *AhAL1*-transgenic plants showed improved survival compared to the WT (Col-0). For each 10 × 10 cm pot, left half was WT, while right half was *AhAL1*-transgenic line; all transgenic lines accompanied with WT were placed in one tray for watering and drought-treatment. (**b**) Survival rate of plants after recovery from severe drought stress. Values are means ± SD from three independent experimental groups (n = 100). (**c**) Performance of the WT (Col-0) and *AhAL1*-transgenic plants under drought condition. The pot size is 8 × 8 cm. (**d**) Plant height at different stages of dehydration and rewatering. Values are means ± SD (n = 20). (**e**) Relative fresh/dry weight of plants after recovery from dehydration compared with the untreated control. Values are means ± SD from three independent experimental groups (n = 18). (**f**) MDA content of plants after drought treatment (n = 3). (**g**) Water loss of detached leaves shown as percentage of the initial fresh weight. Values are means ± SD from four independent experimental groups (12 detached leaves per each group). For all data, asterisks indicate significant differences from the WT (Student’s *t* test; *P < 0.05; **P < 0.01).

The performance of *AhAL1*-transgenic lines under drought stress was also investigated. After stopping watering for two weeks from the end of cotyledon stage, most of WT plants were withered and dead, whereas *AhAL1*-OE plants performed much better, as judged from significantly higher survival rates compared to the WT (Fig. 4a,b). The two *AhAL1*-high-expressing lines 1–5 and 1–10 were most tolerant to drought stress, showing highest survival rates (Fig. 4b). To investigate affection of long-term drought stress on plants vegetative and reproductive growth, well-grown 18-day-old plants were stopped watering for drought treatment. Although the *AhAL1*-OE lines 1–5 and 1–10 showed lower plant height than the WT in normal condition (Supplementary Fig. S11), their performances under drought stress were significantly better than the WT, as judged from higher bolting inflorescence and plant height during both stress and recover stages (Fig. 4c,d). After re-watering for 8 d, the relative fresh weight and relative dry weight per plant were measured and the two parameters were at higher levels in the *AhAL1*-OE plants than that of the WT (Fig. 4e). Water deficit leads to rapid dehydration and membrane damage, so MDA content and water loss rate were measured to reveal the stress status. Without watering for two weeks, the MDA content in *AhAL1*-transgenic plants were significantly lower than that in the WT, implying lower degree of membrane damage in the *AhAL1* transgenic plants (Fig. 4f). The water loss rate was also determined in detached leaves and the values in the *AhAL1* transgenic plants were apparently lower than that in the WT (Fig. 4g). All these results demonstrate that AhAL1 confers enhanced drought tolerance in transgenic Arabidopsis plants.

### AhAL1 enhances ABA responses in transgenic plants

**Figure 5:**
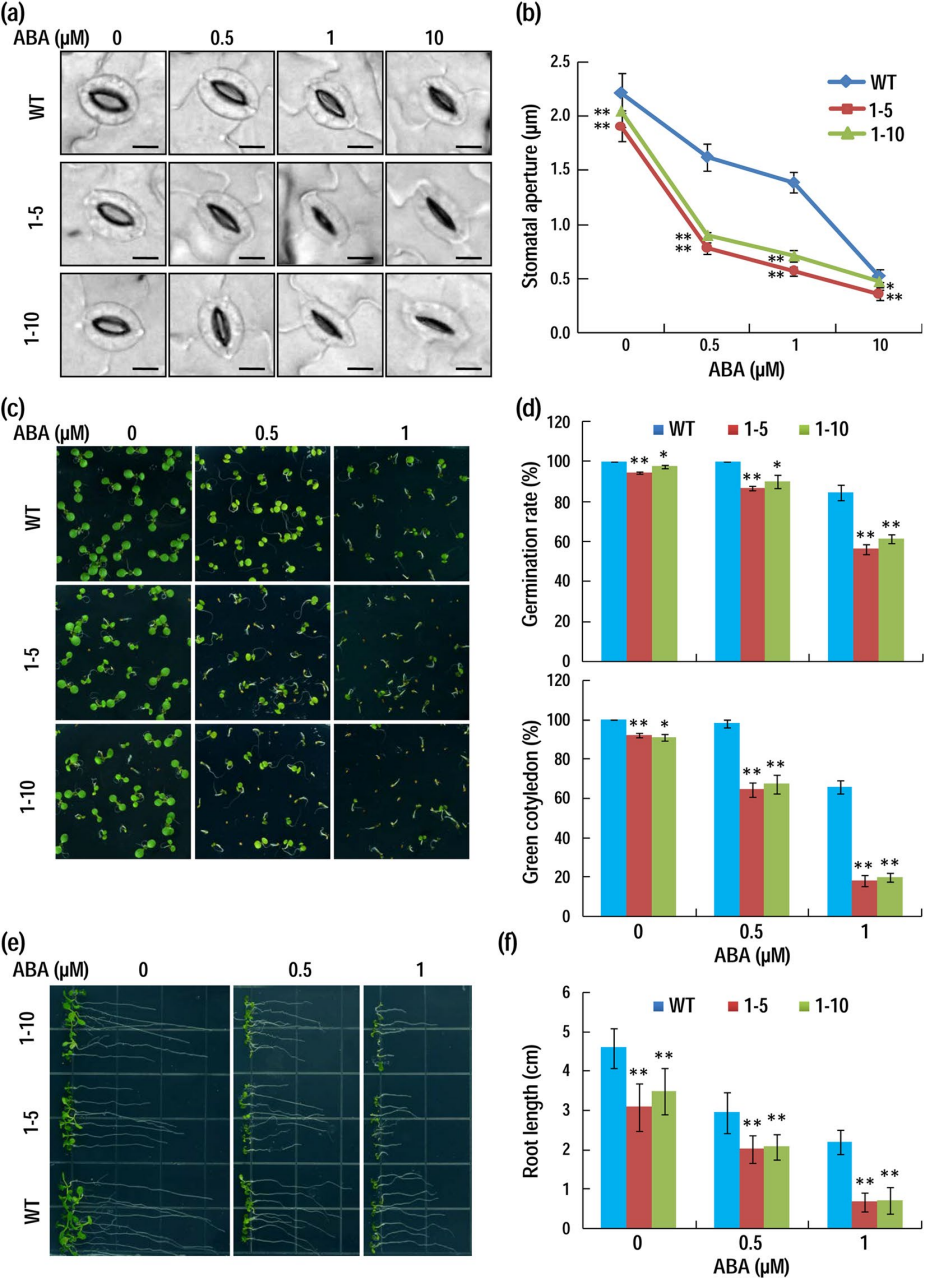
*AhAL1* enhances ABA responses in transgenic plants. (**a**) Leaf stomata closure under treatment with different concentration of ABA (Bar = 5 μm). (**b**) Measurement of stomatal aperture after ABA-treatment for 2 h. Data are means ± SD (n = 30). (**c**) Growth of WT and transgenic seedlings under treatments with different concentrations of ABA. Photographs were taken 5 d after sowing. (**d**) Seed germination assay and quantification of green cotyledons under treatments with different concentrations of ABA. Number of seeds with radicle were counted every half of a day, and germination rates of WT and transgenic lines 4 d-after-sowing were shown. Percentage of seedlings with green cotyledons was quantified 5 d after sowing. (**e**) (**f**) Primary root elongation of WT and transgenic plants under ABA treatment. Primary root length was measured 8 d after planting. For all experiments: Three biological replicates were performed with similar results, and one of them was shown; Significance of data difference between WT and each transgenic line was analyzed by student’s *t* tests (*P < 0.05; **P < 0.01).

Considering that the water loss in *AhAL1*-transgenic plants under water deficit condition was reduced, we speculated that processes related to stomata may be altered. After exposing to light (150 μmol.m^−2^.s^−1^) for 2 h, leaves of transgenic plants exhibited slightly narrower stomatal aperture compared with that of the WT plants (Fig. 5a,b). As an important phytohormone in regulating plant adaptation to drought and salt stress, ABA participates directly in controlling stomatal movements and water transpiration. Then we investigated whether ABA affects stomatal closure in WT and *AhAL1* transgenic plants. It can be seen that the stomata in *AhAL1* transgenic plants are closed more tightly than those in the WT after ABA treatment, especially under 0.5 and 1 µM concentration of ABA (Fig. 5b). This result indicates that AhAL1 promotes ABA-elicited stomatal closure for plant adaptation to water deficit.

We further investigated whether AhAL1 is related to some other ABA-regulated biological processes, including seed germination, seedling establishment and primary root elongation. Although seed germination and cotyledon greening of all genotypes were obviously restrained under ABA treatment, germination rate and green cotyledon rate of *AhAL1* transgenic lines were significantly lower than WT, and the disparities were positively correlated with ABA concentration (Fig. 5c,d). In addition, ABA-elicited hindrance of primary root growth in *AhAL1* transgenic plants was more severe than in WT plants (Fig. 5e,f). It is noteworthy that even without supplement of ABA, all these indexes in *AhAL1* transgenic plants were still significantly lower than in WT plants, indicating somewhat constitutive ABA response in *AhAL1* transgenic plants. These results suggest that introduction of AhAL1 into Arabidopsis plants enhanced ABA response during both germination and post-germination growing stages.

### AhAL1 regulates the expression of ABA- and stress-related genes

**Figure 6:**
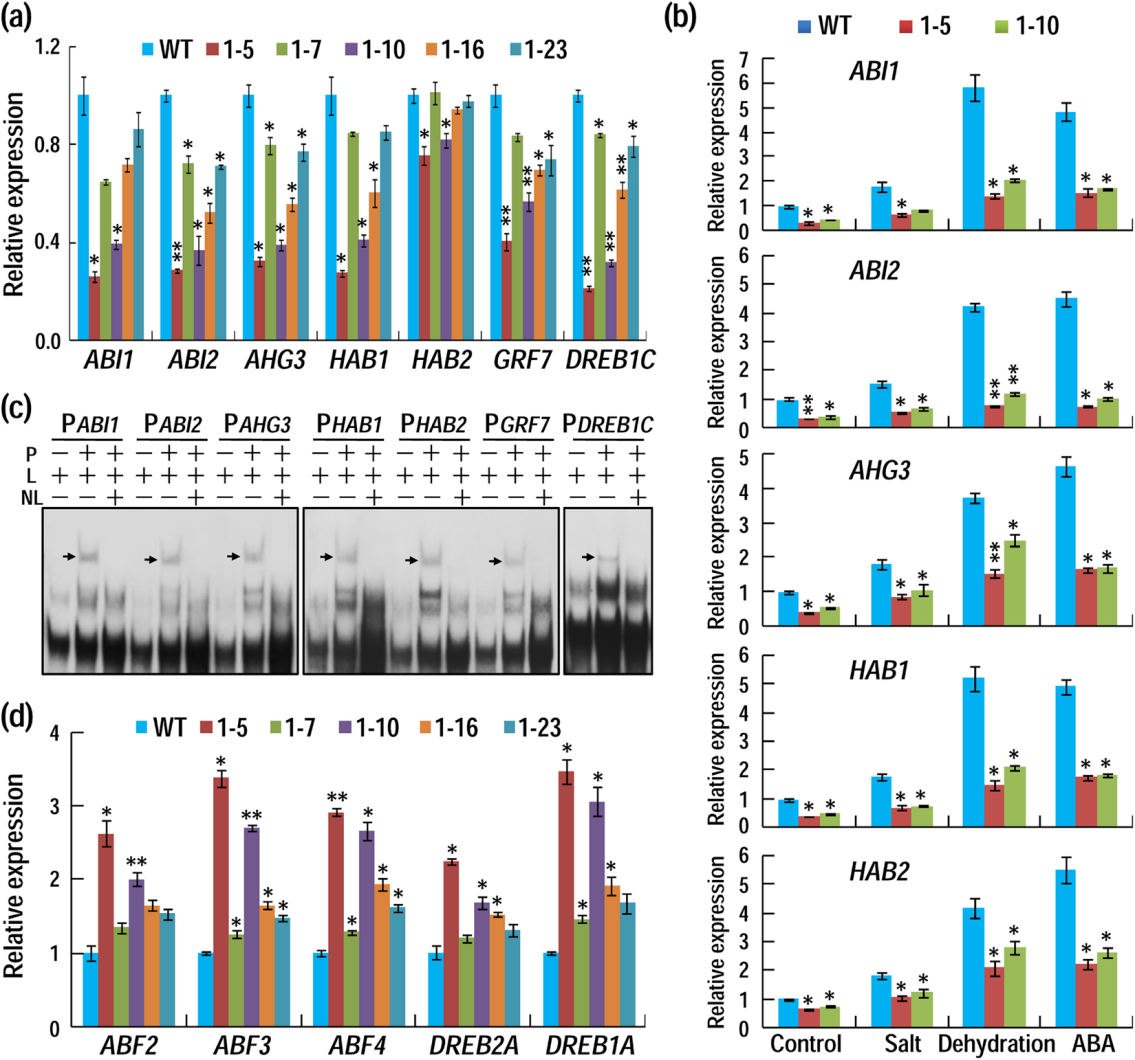
AhAL1 regulates the expression of a series of ABA and stress-responsive genes. (**a**) Identification of AhAL1-downregulated genes by qRT-PCR. (**b**) AhAL1 attenuates stresses (salt, dehydration and ABA)-induced expression of some group-A PP2C-encoding genes. 12-day-old *Arabidopsis* seedlings were treated with 150 mM NaCl, 300 mM mannitol and 50 μM ABA for 6 h respectively, and total RNA was extracted for qRT-PCR analysis. (**c**) Capacity of AhAL1 binding to promoter regions of its downregulated genes revealed by gel-shift assay. P, GST-AhAL1 protein; L, biotin-labeled probes containing one copy of GNGGTG/GTGGNG; NL, unlabeled probe (one hundred fold of the labeled probes). Arrows indicate bands of protein/DNA complexes. Blots cropped from different parts of the same exposure, or from different exposures were made explicit using black dividing lines and white spaces. (**d**) Identification of AhAL1-upregulated genes by qRT-PCR. For all qRT-PCR assay, Arabidopsis *MON1* gene was used as the endogenous control. Values are relative to the expression level in untreated WT plants and shown as means ± SD (n = 3). Student’s *t* tests between WT and each transgenic line were performed (*P < 0.05; **P < 0.01). Three biological replicates were performed with similar results, and one of them was shown.

Since AhAL1 is a transcription repressor and improves stress tolerance possibly though affection of ABA response, we then examined whether the relevant gene expression was altered. We first focused on the putative downstream genes, whose promoter region should contain the cis-element GNGGTG/GTGGNG. As shown in Fig. 6a, the expressions of five group-A protein phosphatase 2 C (PP2C)-encoding genes (*ABI1*, *ABI2*, *AHG3*, *HAB1* and *HAB2*), a *DREB1A* negative regulator gene (*DREB1C*) and a *DREB2A* negative regulator gene (*GRF7*, *GROWTH-REGULATING FACTOR7*) were all decreased in *AhAL1*-transgenic plants, especially in the lines with high expression levels^33–35^. The promoters of these genes all contained at least one core cis-element GNGGTG/GTGGNG (Supplementary Table S3). Further examining the expression of these genes under salt, dehydration and ABA treatments showed that *ABI1*, *ABI2*, *AHG3*, *HAB1* and *HAB2* genes were induced obviously by these stresses in WT plants, whereas in *AhAL1* transgenic plants, the induction was significantly attenuated (Fig. 6b). Gel-shift assay further demonstrated that AhAL1 can specifically bind to promoter regions of these genes (Fig. 6c). Correspondingly, genes encoding ABA-responsive element (ABRE)-binding factors (ABF2, ABF3 and ABF4), *DREB1A* and *DREB2A* were all up-regulated in *AhAL1* transgenic plants (Fig. 6d). These data indicate that AhAL1 may directly bind to the promoters of some negative regulator genes in ABA signaling and inhibit their expression, which then leads to the activation of some ABA/stress-responsive genes to improve plant adaptation to adverse environment.

## Discussion

Researchers have discovered plenty of transcription factors which are involved in plant response and/or tolerance to stress^5–12,23,24,36,37^. Among these factors, the AL/PHD family received relatively less attention. In this study, we identified four *AhAL* genes from the stress-tolerant plant species *A. hortensis*, and found that AhAL1 enhanced salt and drought stress tolerance and ABA response in transgenic Arabidopsis plants. Similarly, some other *AL* genes such as alfalfa *Alfin1*, soybean *GmPHD2* and Arabidopsis *AtAL5* had been reported to improve stress tolerance in transgenic plants^14,21,23,24^. In contrast, constitutively expressing anyone of the other three *AhAL* genes caused plant hypersensitive response to salt stress (Supplementary Figs S5 to S10). A previous study found that overexpression of *AtAL7* decreased plant tolerance to salt stress, while loss-of-function mutation of *AtAL7* promoted root growth under normal and saline conditions, suggesting that *AtAL7* plays a negative role in salt tolerance^27^. Mutation of another Arabidopsis *Alfin-like* gene *AtAL3* also resulted in a moderate increase of plant salt tolerance^27^. Besides, some AL members may be involved in some other biological activities. For example, Arabidopsis AtAL6 is necessary for root hair elongation under phosphate deficient condition^38^. These results indicate functional divergence of different AL members in different plant species.

Genome-wide investigation and expression profiling unraveled that different member within one gene family usually evolved into distinctive expression patterns and cellular localizations for their functional divergence^39,40^. The binding ability of AL proteins to the active histone markers H3K4me3/2 and cis-element GNGGTG/GTGGNG suggests that ALs may function as transcriptional regulators. Actually, our present and previous studies have uncovered that most ALs from soybean, Arabidopsis and *A. hortensis* are likely to be transcription repressors and localized to the nucleus (Fig. 2d)^14,21^. As most transcription regulators should localize in the nucleus to control gene transcription, expression pattern variation may be one of major pathways for function diversifying within one transcription factor family, so does the AL family^40–42^. On one hand, different *AL* genes from different species showed diverse expression levels and organ expression patterns. Although most *AL* genes are universally expressed in vegetative organs, the expression level of alfalfa *Alfin1* in root is much higher than that in leaf, and the six soybean *AL/PHD* genes seem to be more expressed in stem than in root and leaf, while the transcripts of *AhAL1* and *AhAL4* are more abundant in leaf and root than in stem (Fig. 1b)^13,21^. On the other hand, the expression pattern of different *AL* gene in response to environmental stimuli is varied. In alfalfa, salt treatment led to decrease of *Alfin1* mRNA in salt-sensitive line, while in salt-tolerant plants, *Alfin1* mRNA showed no decrease^13^. Most of soybean *AL/PHD* genes were dramatically induced by salt/drought/ABA/cold treatment, but each of them showed distinct expression pattern^21^. In Arabidopsis, most of *AL* genes are inducible under salt, drought and ABA treatment, though with not very high induction levels^14^. Similar to Arabidopsis *AL* genes, *AhAL1* in *A. hortensis* was moderately induced by salt and ABA treatment. However, the expression levels of other three *AhAL* genes were all decreased under salt stress, just like *Alfin1* in salt-sensitive alfalfa plants^13^. To some extent, the contrary expression pattern between *AhAL1* and the other three *AhAL* genes in response to salt stress reflects the opposite role in plant stress response.

In addition to expression diversification, molecular structure variation may also contribute to functional divergence of AL family. AL/PHD proteins contain a conserved N domain at N-terminal end, a PHD domain at C-terminal end and a variable region V domain between them^21^. N domain is mainly responsible for DNA binding, and N domain plus V domain are required for dimerization of homodimers and/or heterodimers between AL/PHD members^21^. A later study further demonstrates that two amino acid residues at positions 34 and 35 of the N domain determine the DNA-binding activity of AL/PHD proteins^14^. From an evolutionary perspective, N domain seems to be more selective than PHD domain during plant evolution, suggesting its primary responsibility for the functional divergence of AL proteins^27^. Nevertheless, most AL family proteins could bind to the active histone markers H3K4me3/2 through the PHD domain and may act as chromatin regulators to control gene expression^20,26^. Exceptionally, the AL3 in Arabidopsis lacks the conserved Tyr residue in its PHD finger and couldn’t bind to H3K4me3/2, and this variation may also contribute to its functional divergence^20^. In the present study, four AL proteins from *A. hortensis* all possess the conserved residues for H3K4me3/2-binding in PHD domain. Therefore, we propose that the contrasting performance of AhAL1 and the other three AhALs in plant adaptation to salinity may come from the molecular variation within N and V domain. However, whether this is the case remains to be further identified. Additionally, we could not exclude the possibility that some AL members may recruit different chromatin remodeling factors and transcription factors, via PHD domain and with affection from V domain, to exert individual functions.

As transcription regulators, AL family should function through controlling the expression of different target genes. Alfalfa Alfin1 was first identified to regulate the expression of *MsPRP2* gene which encodes a putative proline-rich cell wall protein and positively regulate plant salt and drought resistance^23,25,43^. Constitutive expression of *GmPHD2* in Arabidopsis inhibited the expression of *CBF2/DREB1C*, *STRS1*, *STRS2*, *SIS8*, and three other negative regulator genes. Eight genes including *WAK5*, *GLP*, *TPP*, *ABI5*, *MDAR*, and three peroxidase-encoding genes were all upregulated in *GmPHD2*-transgenic plants. Expression changes of these stress-related genes would affect stress signaling, promote ROS scavenging, and finally enhanced plant stress tolerance^21^. In Arabidopsis, AL5 could bind to the promoters of eight target genes (*SHMT7*, *bZIP50*, *CAX1*, *TAC1*, *FAO*, *OFE*, *NAC075* and *WRKY11*) and inhibit their transcription. Knockout mutants of five genes (*TAC1*, *SHMT7*, *FAO*, *OFE* and *CAX1*) all showed increased tolerance to salt, drought and freezing stress^14^. Among these genes, *FAO* encodes a long-chain fatty acid alcohol oxidase and is involved in oxidation of long-chain fatty acids to generate peroxide. Therefore, both GmPHD2 and AL5 may function at least partially through affection of ROS/peroxide production. Arabidopsis AL6 controls the expression of multiple downstream genes including *ETC1*, *NPC4*, *SQD2* and *PS2* to promote root hair elongation under phosphate deficient condition^38^.

In this study, AhAL1 was found to bind promoter regions of *GRF7*, *DREB1C* and several ABA-related group-A PP2C-encoding genes and downregulate their expression. Correspondingly, the expression of *DREB2A*, *DREB1A* and three *ABF* genes were increased in *AhAL1*-transgenic plants. As a result, *AhAL1*-transgenic plants became more sensitive to ABA in stomatal closure, seed germination and primary root elongation (Fig. 5). Similarly, constitutive expression of *GmPHD2* in Arabidopsis enhanced the expression of *ABI5*, a positive regulatory gene in ABA signaling^21^. Moreover, the negative regulator gene *CBF2/DREB1C* was inhibited by both GmPHD2 and AhAL1 in transgenic plants (Fig. 6)^21^. These data indicate that some AL/PHD family proteins like AhAL1 and GmPHD2 may promote ABA and DREB pathway for plant adaptation to environmental stress. It should be noted that although the repression and up-regulation of some ABA and stress-related genes show some proportionality to *AhAL1* expression level (Fig. 6a and d), stress tolerance and ABA response phenotypes are not so proportional (Figs 3 to 5), so does the AhAL1-confered attenuation of ABA induced expression of some group-A PP22C encoding genes (Fig. 6b). These unexpected results suggest that while ABA plays a role in improving stress tolerance in *AhAL1*-transgenic Arabidopsis, their relationship is likely to involve more complex interactions. Under stress, AhAL1 may regulate these genes not directly through ABA-signaling, other ways might also exist. In addition, AhAL1 may associate with some unknown factors to co-regulate the expression of these genes, just like our recent findings on the GmPHD6 in soybean^44^.

In conclusion, constitutive expression of *AhAL1* alters the expression of multiple ABA and stress-related genes, and promotes ABA response and tolerance to salt and drought stress. In contrast, three other *AhAL* genes all conferred salt hypersensitive response in transgenic plants. Our study reveals functional divergence of AL family and identifies *AhAL1* as a candidate gene for genetically improving stress tolerance of crop plants.

## Methods

### Plant materials and stress treatments

Seeds of *A. hortensis* were sown in soil. After germination, seedlings grew at 23 °C under a 16-h-light/8-h-dark photoperiod. After growing for two weeks, the whole seedlings were used for total RNA extraction and *AhALs* gene cloning. To detect the expression pattern of *AhAL* genes under environmental stress, two-week-old seedlings were subjected to various stress treatments, including high salinity, drought, and ABA. For salt (200 mM NaCl) and ABA (100 μM) treatments, the seedlings were carefully pulled out from soil and immersed in the corresponding solutions for various times. The seedlings were also placed on bench (25 °C, in air with 60% humidity) for dehydration (drought). The seedlings in pots were placed at 0 °C for cold treatment. Treatment with water was set as a control. At different treating time, shoot of plants were collected for expression assay of *AhAL* genes. Besides, leaves, roots and stems of 3-week-old *A. hortensis* seedlings were collected for detecting the organ-specific expression pattern of *AhAL* genes. The expression of *AhAL* genes were detected by real-time quantitative reverse transcription PCR (qRT-PCR) with primers listed in Supplementary Table S1.

### Cloning and sequence analysis of *AhAL* genes

To clone *Alfin-like* genes from *A. hortensis*, we performed sequence alignment of multiple AL proteins from alfalfa, Arabidopsis, soybean and rice, and designed a pair of degenerate primers (AhALF1 and AhALR1, Supplementary Table S2) according to the most conserved region in the DUF3594 and PHD domain respectively. With this pair of degenerate primers, we isolated several *AhAL* cDNA fragments by RT-PCR using cDNA reverse-transcribed from the total *A. hortensis* RNA. Based on these partial sequences, the 5′-end and 3′-end of these AhAL cDNAs were obtained using the classical SMART RACE method with primers shown in Supplementary Table S2, and the full length Coding DNA Sequences (CDS) was isolated and cloned into pMD18-T vector (TaKaRa, D103A).

Protein sequence of AhALs was aligned and sequence identities were calculated using the MegAlign (DNASTAR Lasergene 12.3) with Clustal W method. Phylogenetic analysis of the AhALs with ALs from other species was conducted using the MEGA 5.0 program with neighbor-joining method (1000 replicates).

### Subcellular localization analysis of AhALs

Full-length CDS of *AhAL* genes were fused to the 5′-end of *Green Fluorescent Protein* (*GFP*) to generate the pUC-AhAL-GFP constructs^45^. These constructs were transfected into Arabidopsis protoplasts by PEG-CaCl_2_ method^46^. The pUC-GFP vector was transfected as a control. After incubation at 23 °C for 16 h, the GFP fluorescence signal was visualized under laser confocal microscope.

### Transcriptional activity assay of AhALs in Arabidopsis protoplast system

Transcriptional regulatory activity was measured using the Gal4-luciferase (LUC) reporter system in Arabidopsis protoplasts with some modifications^47,48^. For transactivation assay, CDS of *AhAL* genes were ligated to the 3′-end of BD domain-coding sequence in expression vector pRT-BD as effectors^21^. The coding sequence of VP16, a herpes simplex virus (HSV)-encoded transactivator, was constructed into pRT-BD vector as a positive control (pRT-35S-BD-VP16). For transcriptional regulatory activity assay, CDS of *AhAL* genes were inserted into pRT107 vector under the control of 35 S promoter, and these constructs plus pRT-35S-BD-VP16 were used as effectors. The 35 S promoter-motivated *AtACTIN7* was used as a non-interaction control^14^. The effector, reporter and internal control (pTRL) were co-transfected into Arabidopsis protoplasts using PEG-CaCl_2_ method. After incubation at 23 °C for 16 h, LUC activity was detected using the Promega dual-luciferase^®^ reporter assay system and a GloMax^®^ 20/20 luminometer (Promega).

### Electrophoretic mobility shift assay (EMSA)

To obtain GST-AhAL proteins, corresponding CDS were ligated into pGEX-6P-1 vector and expressed in *E. Coli* (BL21). Proteins were purified using glutathione Sepharose 4B (GE Healthcare). A pair of oligonucleotides containing two copies of GTGGAGA element was synthesized as shown in Fig. 4, and formed into double-stranded DNA in 50 mM NaCl solution by heating at 75 °C for 10 min and slowly cooling down to room temperature. The EMSA assay was performed as described previously^49^.

EMSA were used to detect whether AhAL1 could bind to the promoter regions of downregulated genes in *AhAL1*-transgenic lines. Core cis-element GNGGTG/GTGGNG in the promoter regions were identified and listed in Supplementary Table S3. Then, probes contain one copy of GNGGTG/GTGGNG element closest to ATG were synthesized and labeled with biotin at 5′-terminus for EMSA assay (Supplementary Table S4). Detailed procedures were according to the instructions of LightShift^®^ Chemiluminescent EMSA Kit (Thermo SCIENTIFIC).

### Generation of *AhALs*-transgenic plants and their performance under stress

The coding regions of *AhAL* genes were inserted into pBIN438 vector under the control of 35 S promoter for constitutive expression in Arabidopsis. Agrobacterium-mediated transformation of Arabidopsis was performed using the floral dip method^50^. Transgenic lines with different expression level of each transgene were selected from dozens of lines and the homozygous transgenic seeds (T3 generation) were used for further studies.

For germination assay, 50–100 seeds per genotype were sown on half-strength Murashige and Skoog medium (1/2 MS) plates containing different concentrations of NaCl and placed at 23 °C under 16-h-light/8-h-dark photoperiod for germination. Seeds showing radicle emergence were counted each day for calculation of germination rate. For salt tolerance assay, 5-day-old seedlings growing on 1/2 MS were transplanted to NaCl-containing 1/2 MS for stress treatment, and their performance under stress was observed each day. After treatment for about one week, obvious phenotypic changes were observed and recorded by photography. For soil salinity tolerance assay, 12-day-old seedlings growing in soil were watered with 250 mM NaCl solution to soil capacity. After about two weeks, the majority of WT plants were withered and dead, and then the salinity soil was dialyzed against water to remove most of salts for plant growth recovery. After recovery for 10 d, photographs were taken and survival rates were calculated. To determine the cell membrane integrity of plant under salinity, relative electrolyte leakage was measured as described^51^.

For measurement of drought-tolerance ability of *AhAL1*-transgenic plants, watering for seedlings growing in soil were stopped from the end of cotyledon stage. After about two weeks, most of WT plants were totally withered and dead, and then all plants were re-watered for recovery and survival rates were calculated. To investigate affection of long-term drought stress on plants vegetative and reproductive growth, well-grown 18-day-old plants were withheld from water for drought treatment. When contrasting stress phenotypes between *AhAL1*-transgenic plants and wild plants appeared, plants were re-watered for growth recovery. After recovery for 8 d, plant fresh weight and dry weight were measured. During drought stress and recovery stage, plant height was measured. To determine the cell membrane damage of plant under dehydration, malonaldehyde (MDA) content was measured as described previously^52^. In addition, water loss of detached leaves was measured as described^53^.

### Observation of stomatal aperture under ABA treatment

Measurement of ABA-mediated stomatal closure was performed as described previously^54^. Detached rosette leaves from 18-day-old plants were floated in solutions containing 20 mM KCl, 50 μM CaCl_2_, 10 mM MES-Tris, pH 6.15 and exposed to light (150 μmol m^−2^ s^−1^) for 2 h. Then, ABA was added at different concentrations (0, 0.5, 1 and 10 μM respectively) to induce stomatal closure. After treatment for 2 h, stomatal apertures were measured as described^55^.

### Phenotypic analysis of ABA responses during germination and post-germination stages

For germination test, 60 seeds per genotype were sown on 1/2 MS medium plates containing different concentrations of ABA. Seeds showing radicle emergence were counted every half of a day for calculation of germination rate. For post-germinative root growth assay, germinated seeds were sown on 1/2 MS medium plates containing different concentrations of ABA. Primary root lengths of seedlings were measured 8 d after planting.

### Real-time quantitative reverse transcription PCR

Within this study, qRT-PCR was used to detect transcript level of *AhAL* genes and downstream genes in various plant materials. An *actin7* (*ACT7*) homolog gene was cloned from *A. hortensis* using RT-PCR with a pair of degenerate primers (ActinF1 and ActinR1) shown in Supplementary Table S5 and used as the endogenous control of qRT-PCR in *A. hortensis*. Arabidopsis *ACT2* and *MONENSIN SENSITIVITY1* (*MON1*) gene was used as endogenous controls in Arabidopsis plant materials^56^. All primers used in qRT-PCR detection were listed in Supplementary Table S1.

## Electronic supplementary material

Supplementary Figures and Tables
