# Supplementary material for: An Alfin-like gene from Atriplex hortensis enhances salt and drought tolerance and abscisic acid response in transgenic Arabidopsis
Source: Sci Rep. 2018 Feb 9;8:2707. doi: 10.1038/s41598-018-21148-9 (PMC5807399; doi:10.1038/s41598-018-21148-9)
Supplement: Supplementary file 1 — Supplementary Figures and Tables [file 41598_2018_21148_MOESM1_ESM.pdf]

An *Alfin-like* gene from *Atriplex hortensis* enhances salt and drought tolerance and abscisic acid response in transgenic Arabidopsis

Jian-Jun Tao[1], Wei Wei[1], Wen-Jia Pan[1], Long Lu[1], Qing-Tian Li[1], Jin-Biao Ma[2], Wan-Ke Zhang[1], Biao Ma[1], Shou-Yi Chen[1*], Jin-Song Zhang[1*]

[1] State Key Lab of Plant Genomics, Institute of Genetics and Developmental Biology, Chinese Academy of Sciences, Beijing 100101, China
[2] Key Laboratory of Biogeography and Bioresource in Arid Land, Xinjiang Institute of Ecology and Geography, Chinese Academy of Sciences, Urumqi, Xinjiang, China
[*] To whom correspondence should be addressed. E-mail: sychen@genetics.ac.cn, jszhang@genetics.ac.cn; Fax: +86-10-64806595

(a)

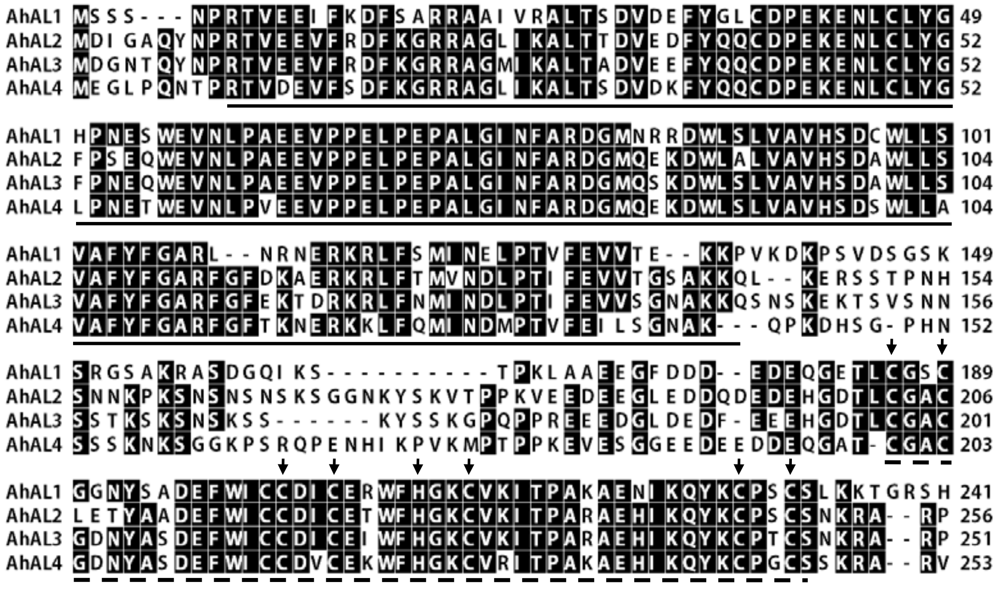

(b)

Percent Identity (upper right) / Divergence (lower left)

	1	2	3	4		
1		61.8	62.2	59.3	1	AhAL1
2	41.7		77.3	65.6	2	AhAL2
3	42.0	21.3		70.1	3	AhAL3
4	46.2	40.1	30.8		4	AhAL4
	1	2	3	4		

Supplementary Figure S1. Sequence comparison of the four AhAL proteins. (a) Sequence alignment using the clustal w method from Lasergene.v7.1. (b) Sequence identity values from multiple sequence alignments. Conserved amino acids are shaded in black. The conserved N domain (DUF3594) and PHD domain are labeled by straight and dashed lines respectively. The conserved C4HC3 residues in PHD finger are indicated by arrows.

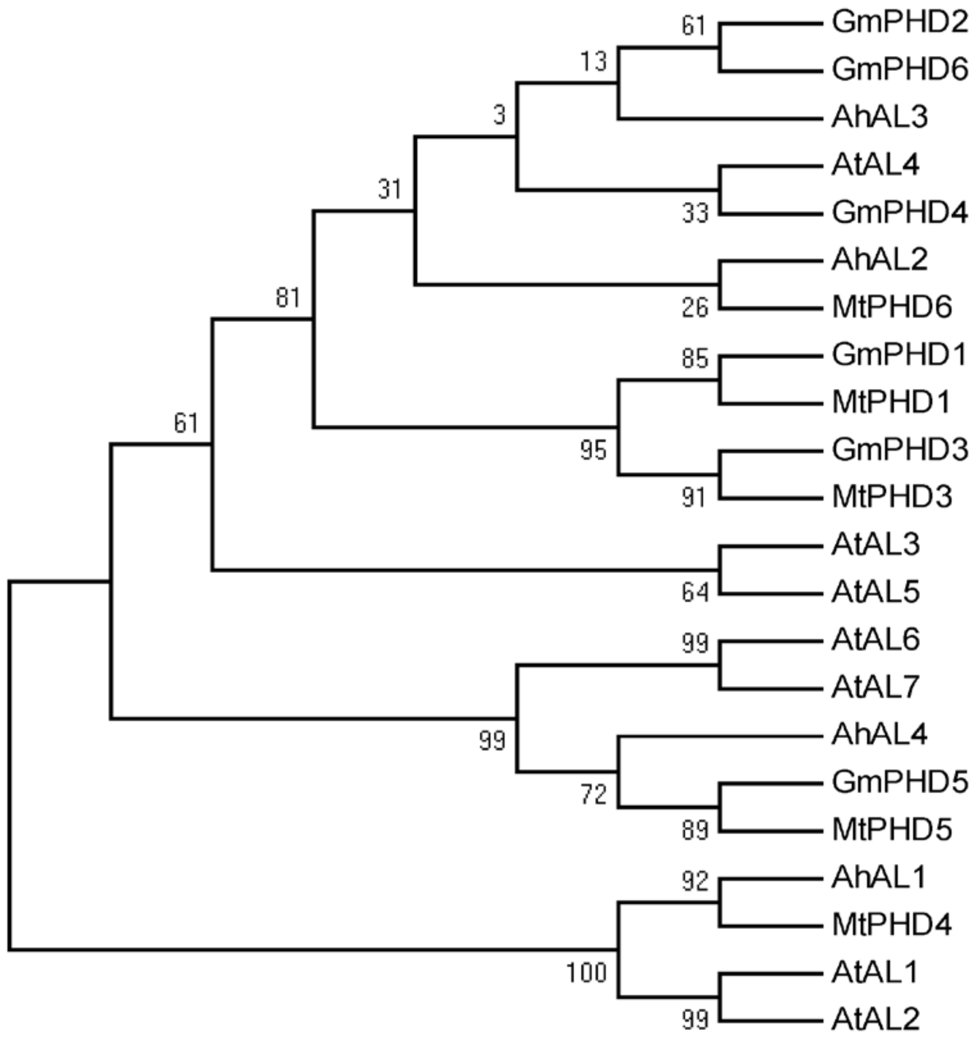

Supplementary Figure S2. Cluster analysis of ALs from different plant species using MEGA 5.0. Numbers on the figure are bootstrap values. AL sequences are from *Atriplex hortensis* (AhALs), *Medicago truncatula* (MtALs), *Glycine max* (GmPHDs) and *Arabidopsis thaliana* (AtALs). Accession numbers are as following: AhAL1 (KU933956), AhAL2 (KY322832), AhAL3 (KY322833), AhAL4 (KY322834); MtPHD1 (ABJ99759.1), MtPHD3 (XP_003597552.1), MtPHD4 (ABJ99761.1), MtPHD5 (ABJ99762.1), MtPHD6 (ABJ99763.1); GmPHD1 (NP_001237921.1), GmPHD2 (NP_001237834.1), GmPHD3 (NP_001237851.1), GmPHD4 (NP_001237867.1), GmPHD5 (NP_001237885.1), GmPHD6 (NP_001237904.1); AtAL1 (NP_196180.1), AtAL2 (NP_187729.1), AtAL3 (NP_189865.1), AtAL4 (NP_197993.1), AtAL5 (NP_197551.2), AtAL6 (NP_178351.1), AtAL7 (NP_172903.1).

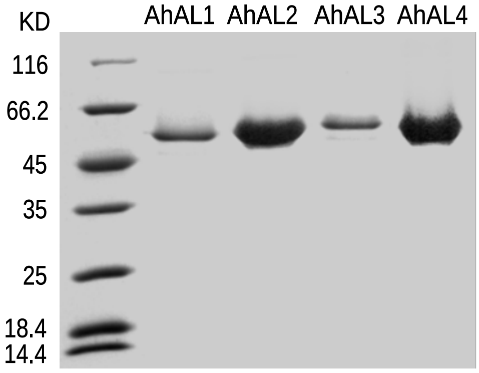

Supplementary Figure S3. Gel electrophoresis of the purified GST-AhAL fusion proteins. Purified GST-AhAL proteins were electrophoresed on 12% SDS-PAGE gel. The leftmost lane is protein markers. Arrows indicates the bands of proteins.

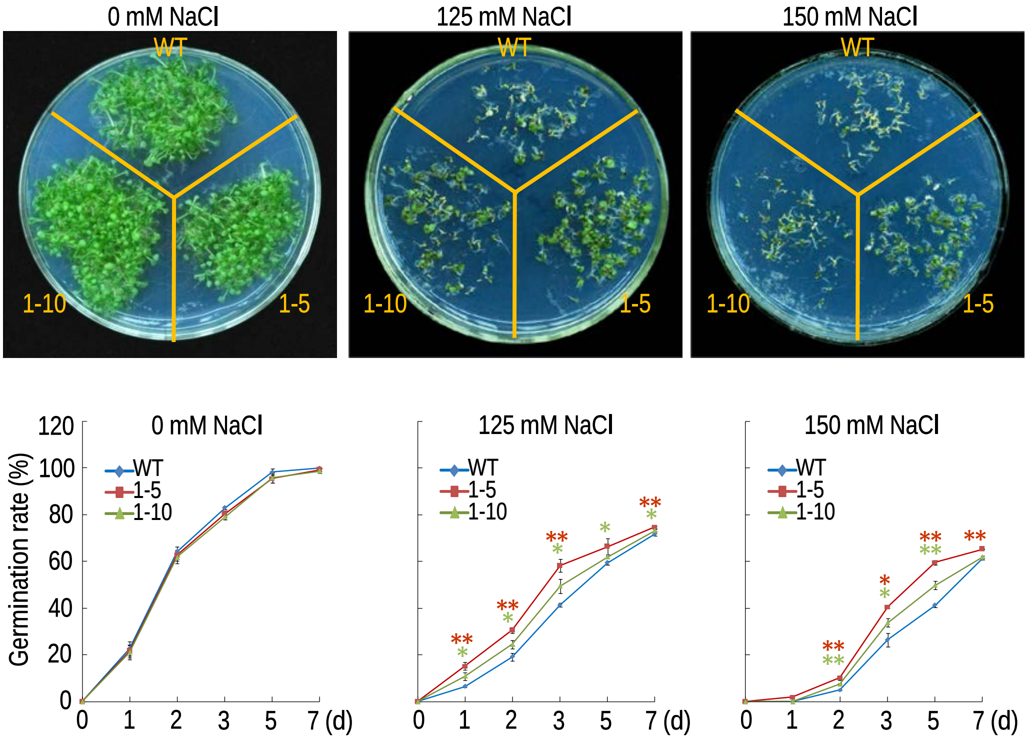

Supplementary Figure S4. Introduction of *AhAL1* into Arabidopsis promoted seed germination under salt stress. Sterilized wild type and transgenic seeds were stratificated at 4 °C in dark for 3 d, and sowed on 1/2 MS medium containing different concentration of NaCl. Seeds germinated each day after stratification were counted and the germination rate was calculated. Data shown are means ± SD from three independent sample groups. Colored asterisks indicate significant difference between corresponding transgenic line and WT (Student's *t* test; *, $P < 0.05$; **, $P < 0.01$).

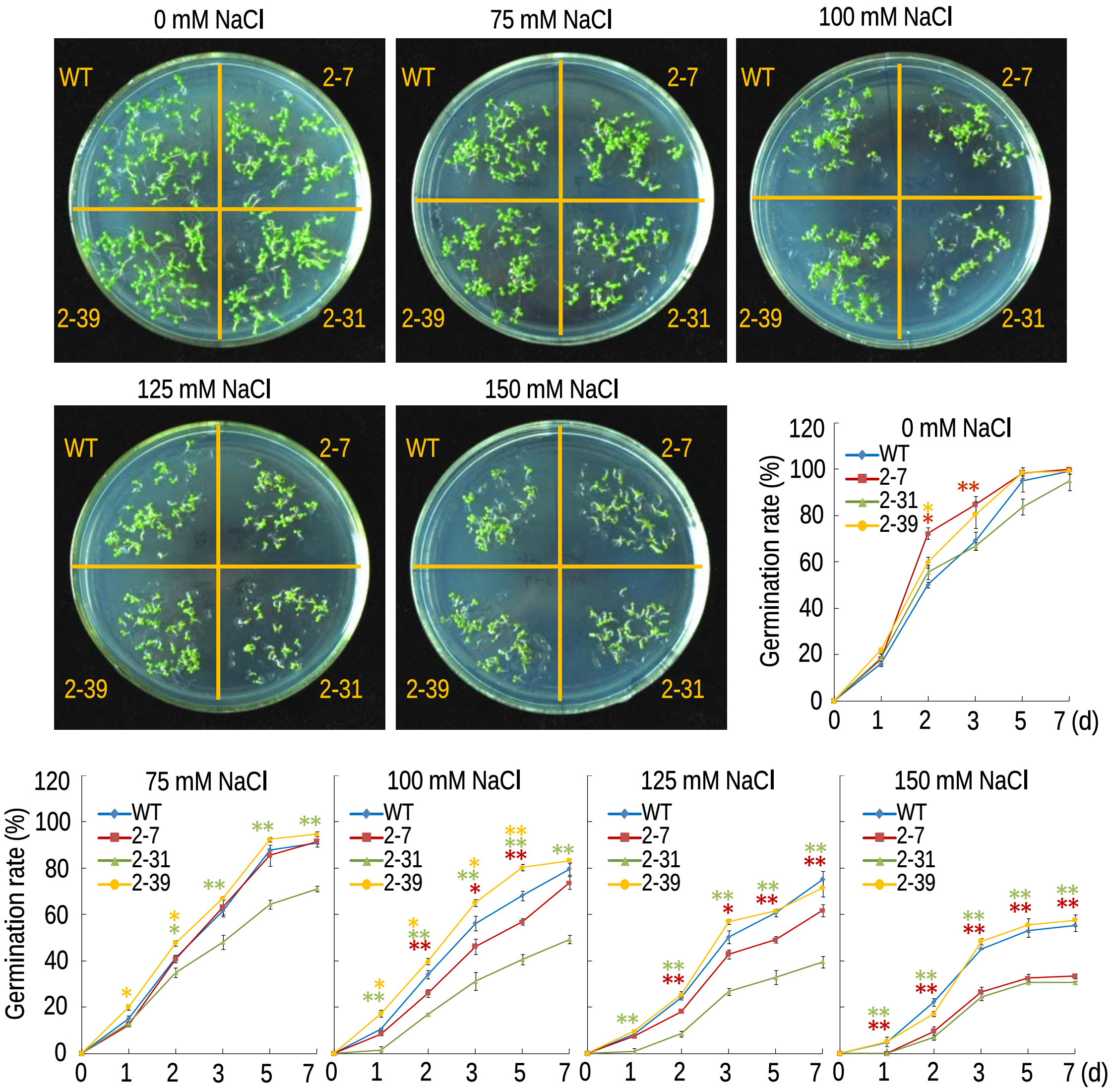

Supplementary Figure S5. Introduction of *AhAL2* into Arabidopsis decreased seed germination under salt stress. Sterilized wild type and transgenic seeds were stratificated at 4 °C in dark for 3 d, and sowed on 1/2 MS medium containing different concentration of NaCl. Seeds germinated each day after stratification were counted and the germination rate was calculated. Data shown are means ± SD from three independent sample groups. Colored asterisks indicate significant difference between corresponding transgenic line and WT (Student's *t* test; *, $P < 0.05$; **, $P < 0.01$).

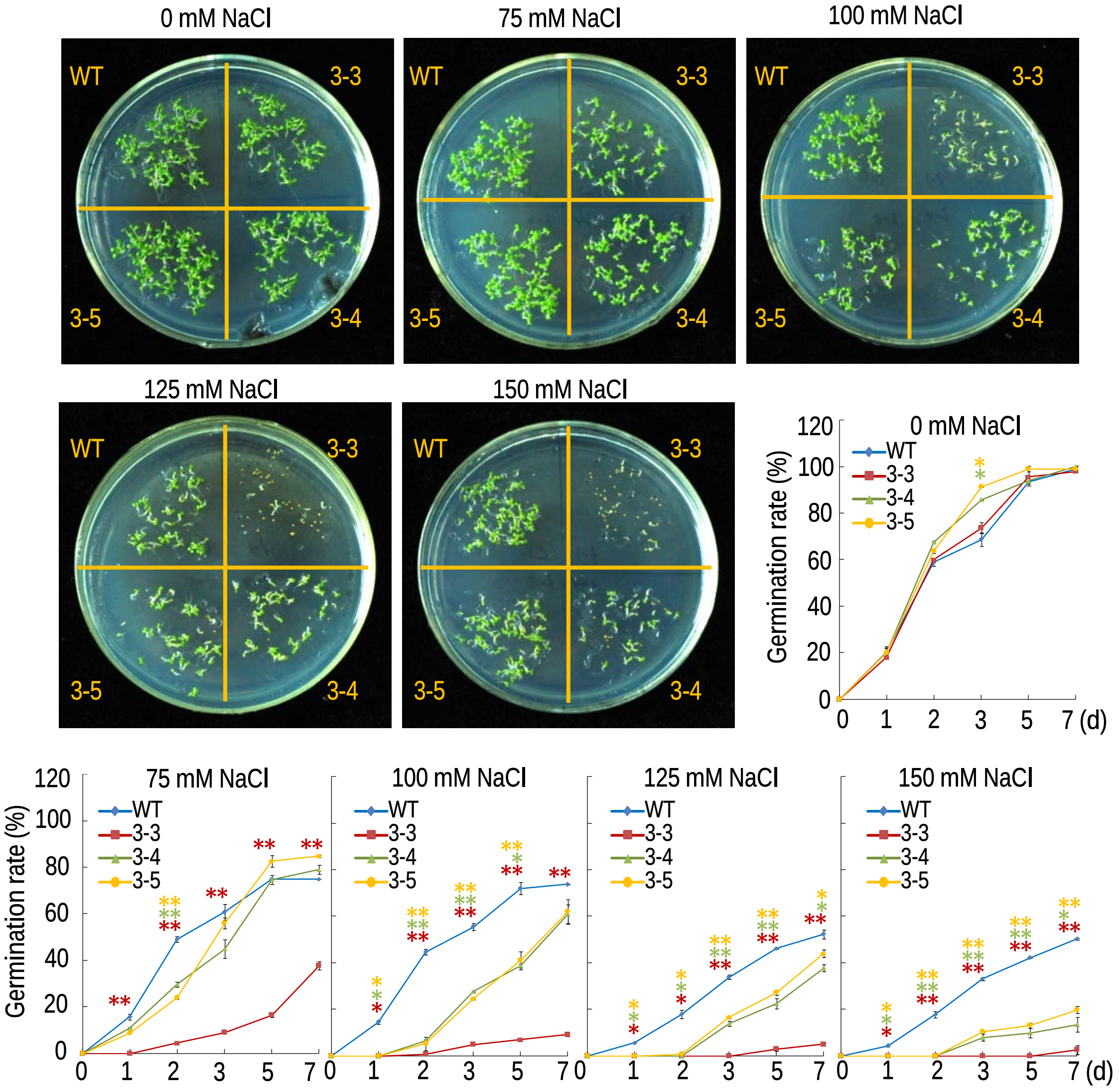

Supplementary Figure S6. Introduction of *AhAL3* into Arabidopsis decreased seed germination under salt stress. Sterilized wild type and transgenic seeds were stratificated at 4 ^{o}C in dark for 3 d, and sowed on 1/2 MS medium containing different concentration of NaCl. Seeds germinated each day after stratification were counted and the germination rate was calculated. Data shown are means ± SD from three independent sample groups. Colored asterisks indicate significant difference between corresponding transgenic line and WT (Student's *t* test; *, $P < 0.05$; **, $P < 0.01$).

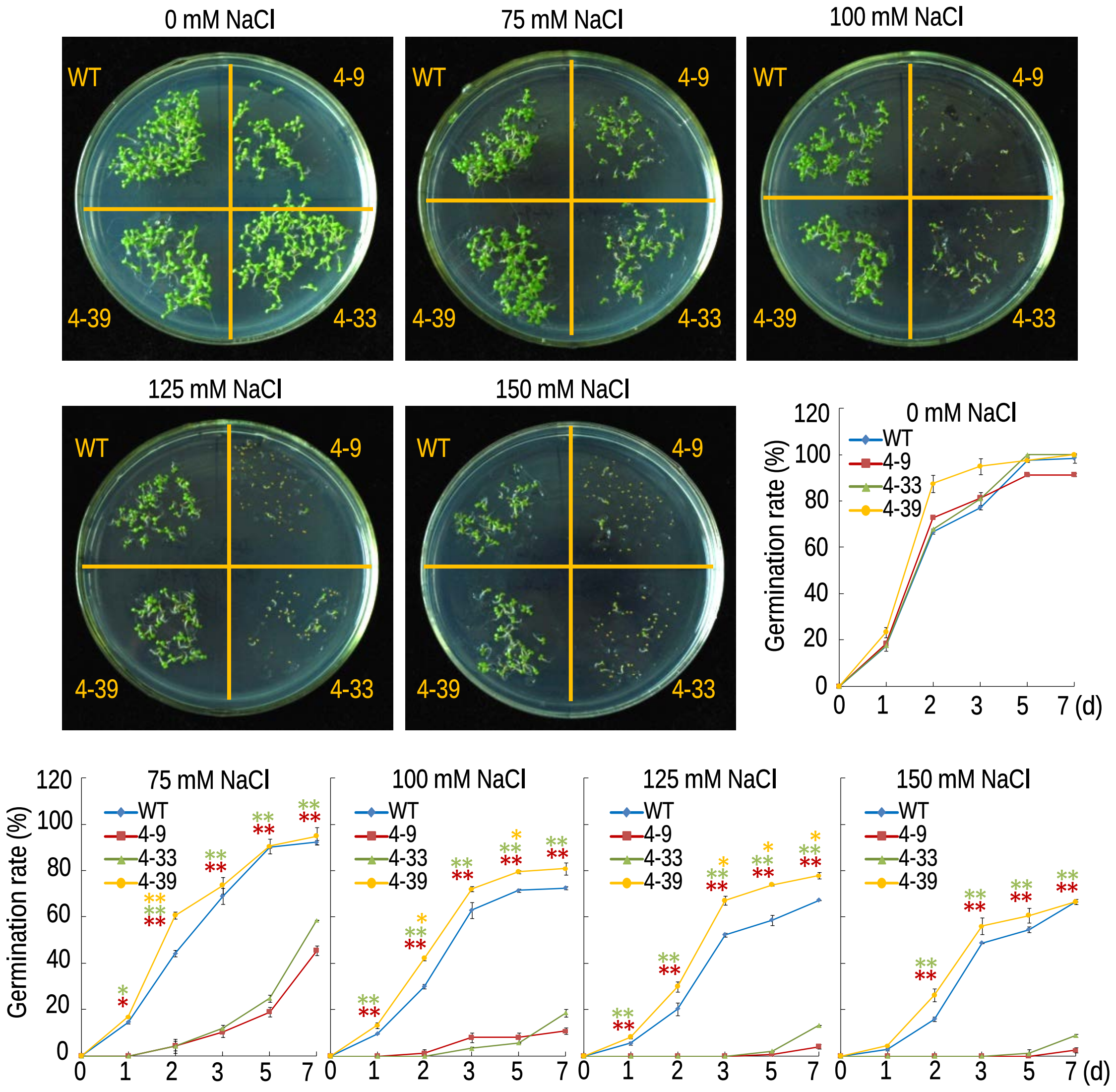

Supplementary Figure S7. Introduction of *AhAL4* into Arabidopsis decreased seed germination under salt stress. Sterilized wild type and transgenic seeds were stratificated at 4 °C in dark for 3 d, and sowed on 1/2 MS medium containing different concentration of NaCl. Seeds germinated each day after stratification were counted and the germination rate was calculated. Data shown are means ± SD from three independent sample groups. Colored asterisks indicate significant difference between corresponding transgenic line and WT (Student's *t* test; *, P < 0.05; **, P < 0.01).

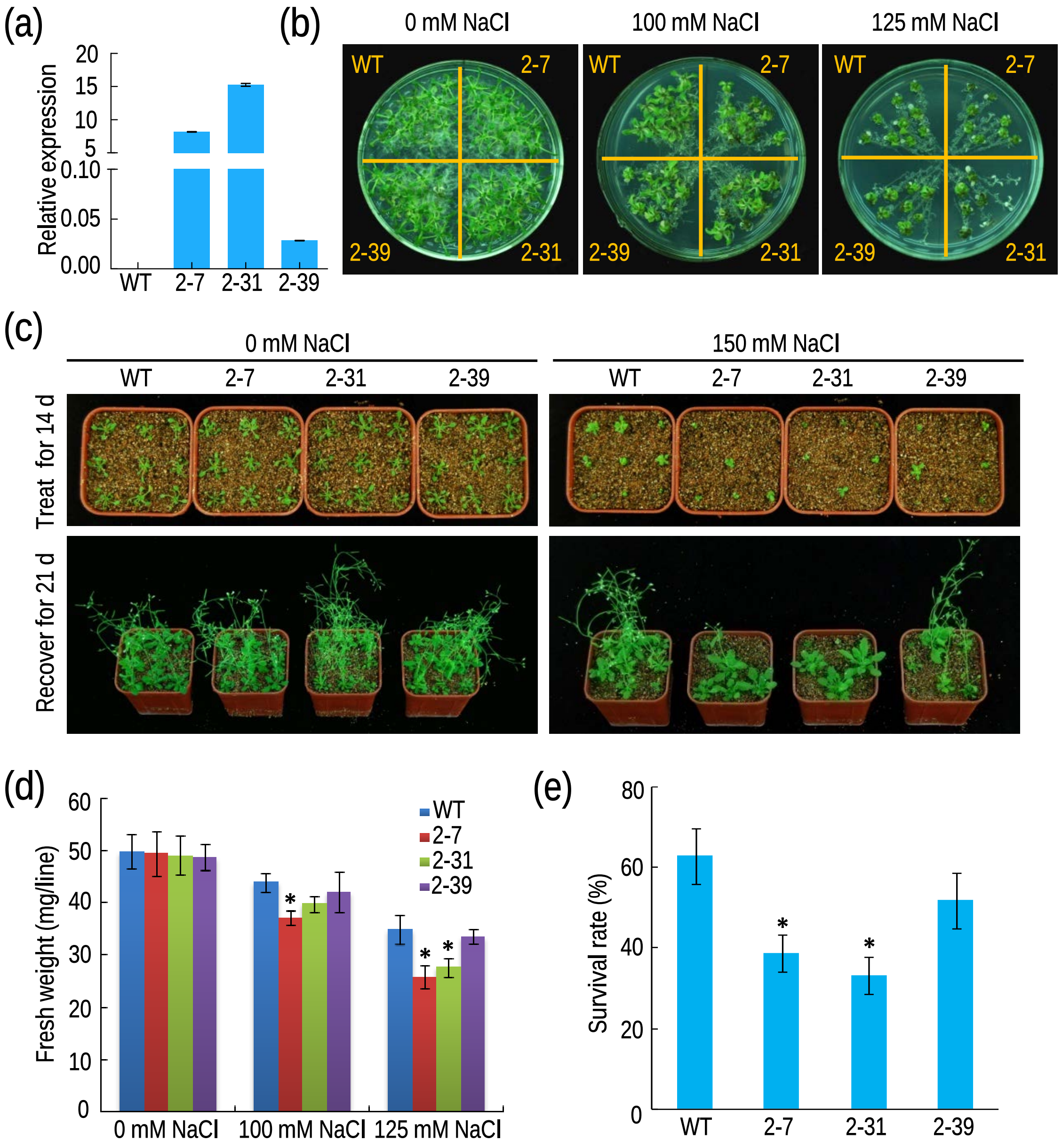

Supplementary Figure S8. Performance of *AhAL2*-transgenic Arabidopsis plants under salt stress. (a) Transcripts level of *AhAL2* in WT and the selected *AhAL2* transgenic lines. Arabidopsis *actin 2* gene *AtACT2* was used as the endogenous control. Values are means ± SD (n = 3). (b) Plant phenotypes after salt treatment on 1/2 MS medium. (c) Performance of plants growing in soil under salt treatment and recovery from stress. The pot size is 8 × 8 cm. (d) Fresh weight of plants after salt treatment. Values are means ± SD from three independent experimental groups (27 plants per each group). (e) Survival rate of plants after recovery from salt stress. Values are means ± SD from three independent experimental groups (27 plants per each group). For all data, asterisks indicate significant differences from the WT (Student's t test; *, $P < 0.05$).

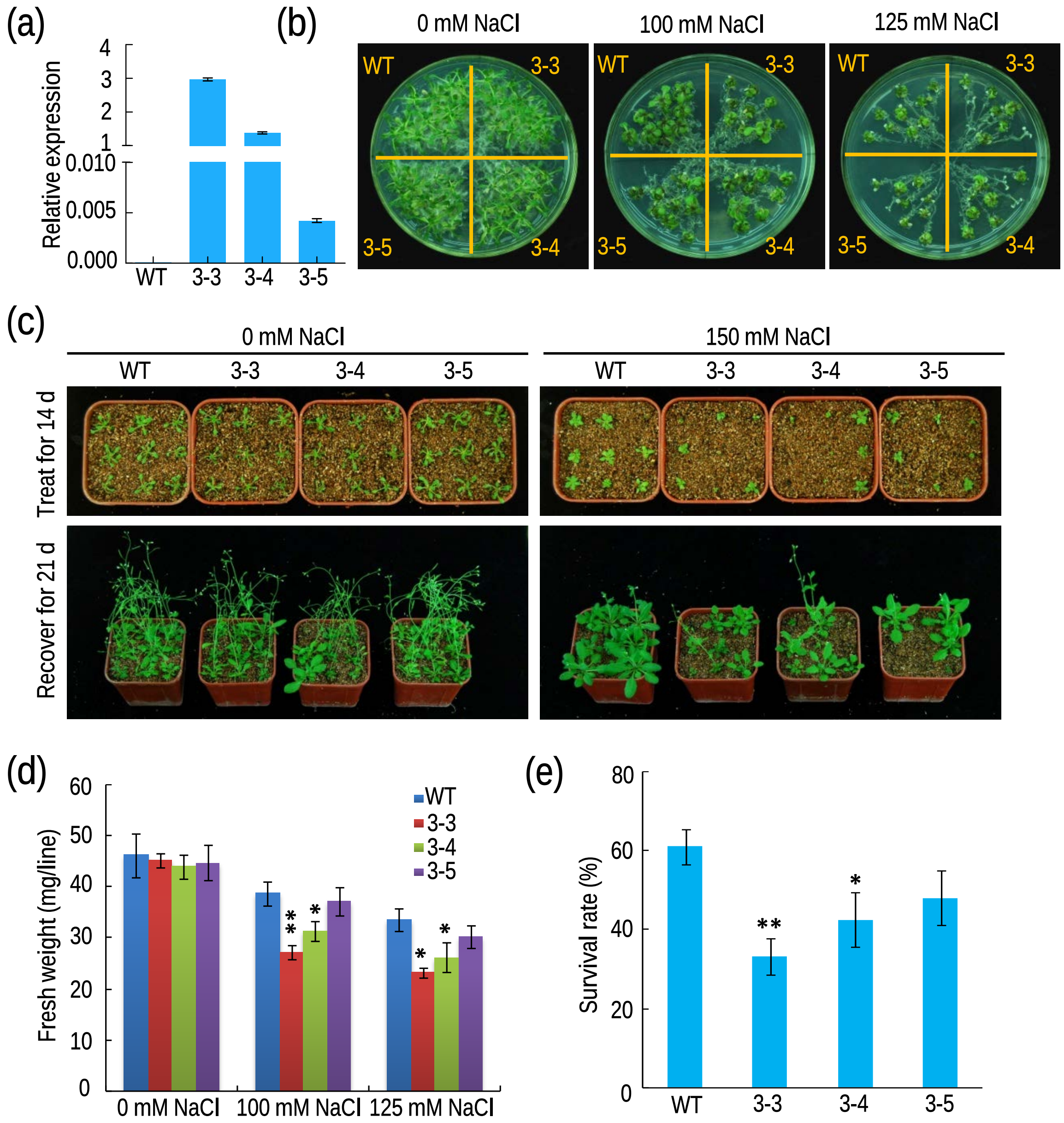

Supplementary Figure S9. Performance of *AhAL3*-transgenic Arabidopsis plants under salt stress. (a) Transcripts level of *AhAL3* in WT and the selected *AhAL3* transgenic lines. Arabidopsis *actin 2* gene *AtACT2* was used as the endogenous control. Values are means ± SD (n = 3). (b) Plant phenotypes after salt treatment on 1/2 MS medium. (c) Performance of plants growing in soil under salt treatment and recovery from stress. The pot size is 8 × 8 cm. (d) Fresh weight of plants after salt treatment. Values are means ± SD from three independent experimental groups (27 plants per each group). (e) Survival rate of plants after recovery from salt stress. Values are means ± SD from three independent experimental groups (27 plants per each group). For all data, asterisks indicate significant differences from the WT (Student's t test; *, $P < 0.05$; **, $P < 0.01$).

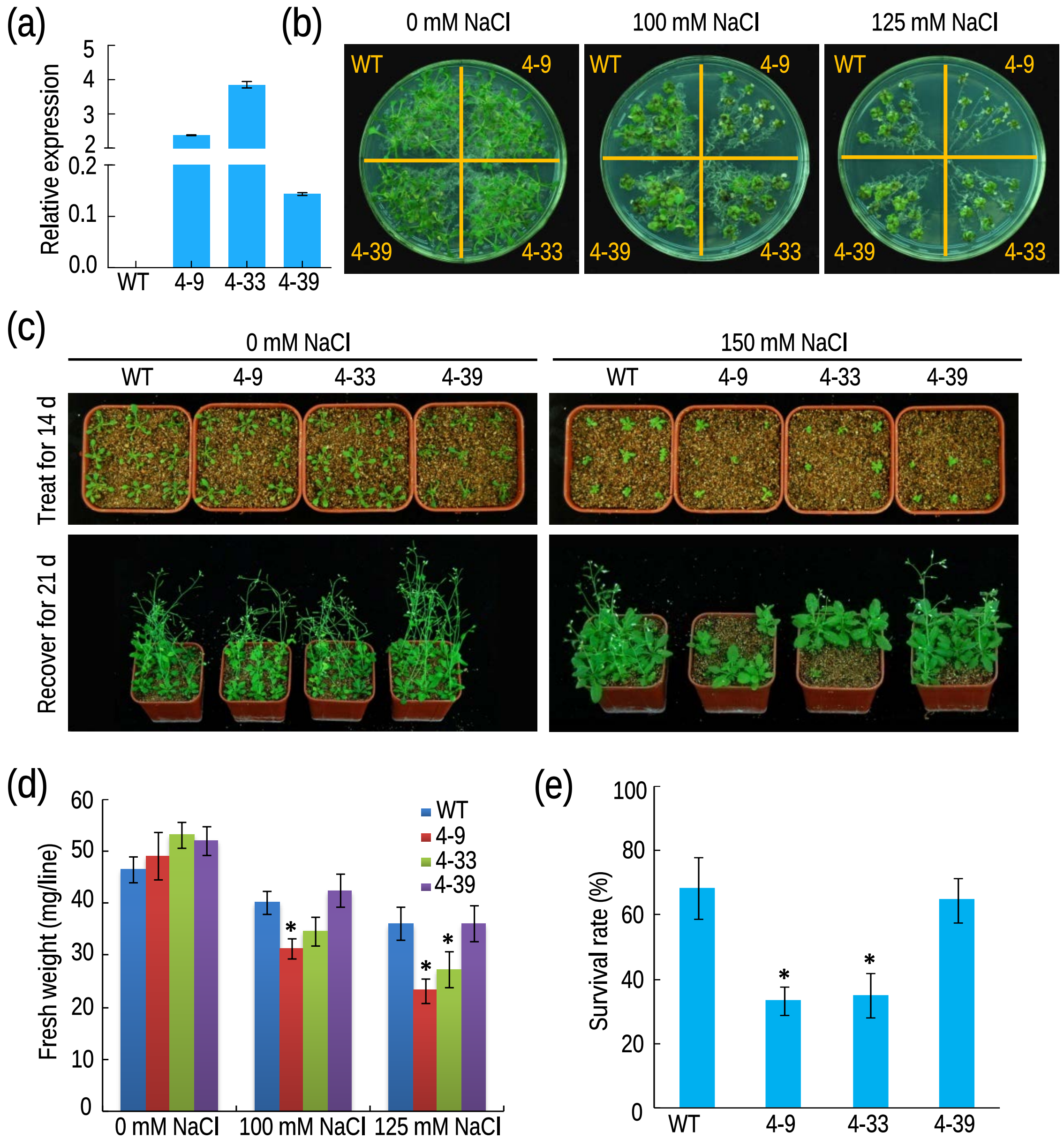

Supplementary Figure S10. Performance of *AhAL4*-transgenic Arabidopsis plants under salt stress. (a) Transcripts level of *AhAL4* in WT and the selected *AhAL4* transgenic lines. Arabidopsis *actin 2* gene *AtACT2* was used as the endogenous control. Values are means ± SD (n = 3). (b) Plant phenotypes after salt treatment on 1/2 MS medium. (c) Performance of plants growing in soil under salt treatment and recovery from stress. The pot size is 8 × 8 cm. (d) Fresh weight of plants after salt treatment. Values are means ± SD from three independent experimental groups (27 plants per each group). (e) Survival rate of plants after recovery from salt stress. Values are means ± SD from three independent experimental groups (27 plants per each group). For all data, asterisks indicate significant differences from the WT (Student's t test; *, $P < 0.05$).

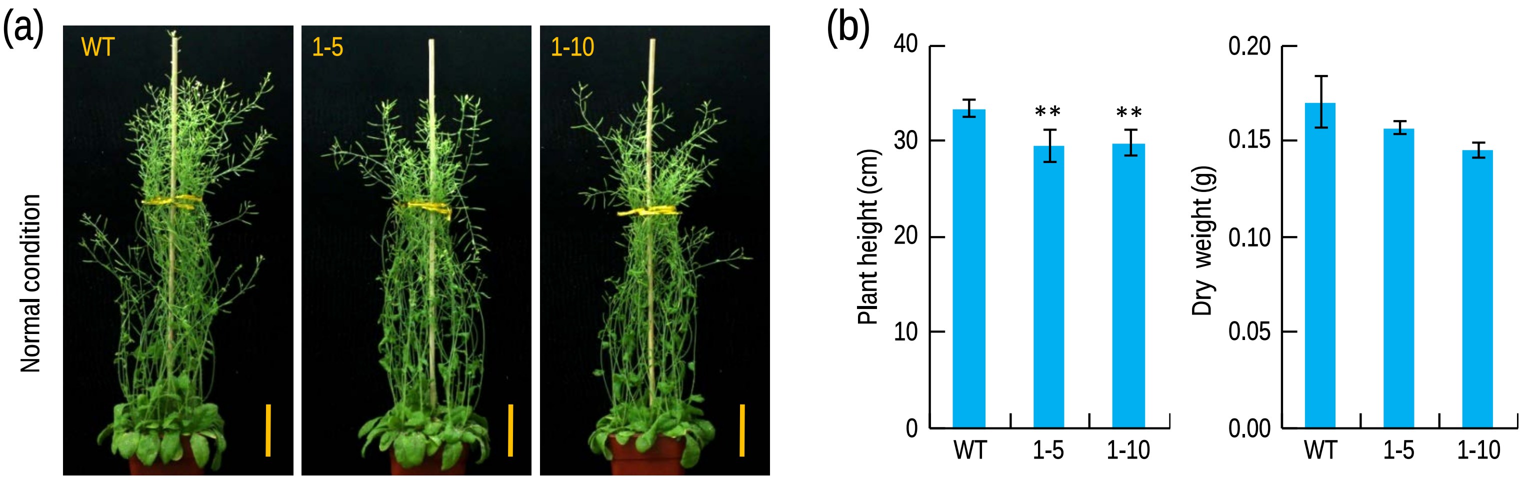

Supplementary Figure S11. Constitutive expression of *AhAL1* in Arabidopsis mildly inhibits plants late growth. (a) Performance of the WT (Col-0) and *AhAL1*-transgenic plants growing in normal condition (Bar = 5 cm). (b) Plant height and biomass (dry weight) of the WT and transgenic plants. Values are means ± SD from three independent experimental groups (n = 18). Asterisks indicate significant differences from the WT (Student's t test; **, $P < 0.01$).

Supplementary Table S1. Primers used for qRT-PCR analysis.

Primer name	Sequence (5→3’)	Gene	Accession Number or Locus
qRT-AhAL1F1	TATTTGCGAGAGGTGGTTCC	*AhAL1*	KU933956
qRT-AhAL1R1	GCCCTGTCTTCTTCAAGCTG		
qRT-AhAL2F1	TGTTCACAGTGATGCCTGGT	*AhAL2*	KY322832
qRT-AhAL2R1	AGCAGAGCCAGTCACAACCT		
qRT-AhAL3F1	CCAAATGAGCAGTGGGAAGT	*AhAL3*	KY322833
qRT-AhAL3R1	AGCCAGTCCTTTGATTGCAT		
qRT-AhAL4F1	AAATGCCTACACCACCGAAG	*AhAL4*	KY322834
qRT-AhAL4R1	CCATTTTTCGCACACATCAC		
qRT-AhactinF1	CTGCTGGTATCCACGAGACT	*Ahactin7*	KY322835
qRT-AhactinR1	GCAATGCCAGGGAACATAGT		
qRT-ABI1F1	GTCGAGATCCATTGGCGATAG	*AtABI1*	AT4G26080
qRT-ABI1R1	CTTGCCATCTCACACGCTTC		
qRT-ABI2F1	GAATTGCGGCGACTCTAGG	*AtABI2*	AT5G57050
qRT-ABI2R1	GGTATCTATCGCCAATGGATCTTG		
qRT-AHG3F1	GCCATGTCTAGAGCAATTGGTGAT	*AtAHG3*	AT3G11410
qRT-AHG3R1	CAGCACCTCGAAGGCACAT		
qRT-HAB1F1	TGTCTAGGTCCATCGGTGACAG	*AtHAB1*	AT1G72770
qRT-HAB1R1	TCGCAGACTTCTTGGTTGTTCAT		
qRT-HAB2F1	CCAGGTCCATCGGTGATCAAT	*AtHAB2*	AT1G17550
qRT-HAB2R1	TTGTGCCAAGCCAAGATCC		
qRT-GRF7F1	GGCTTGCTCCGATGTCTACC	*AtGRF7*	AT5G53660
qRT-GRF7R1	CGAGCTTGGAGTGGGAGTCT		
qRT-DREB1CF1	ACGACGGATGCTCATGGTCT	*AtDREB1C*	AT4G25470
qRT-DREB1CR1	ACGGCGACGGTAAAAGCAT		
qRT-DREB1AF1	GATCATGGCTTCGACATGGAG	*AtDREB1A*	AT4G25480
qRT-DREB1AR1	AGCCAACAAACTCGGCATCT		
qRT-DREB2AF1	TGACCTAAATGGCGACGATG	*AtDREB2A*	AT5G05410
qRT-DREB2AR1	CTCGAGCTGAAACGGAGGTATT		
qRT-ABF2F1	TTCACGGCAGACCAAACAAT	*AtABF2*	AT1G45249
qRT-ABF2R1	CCATGATCCTTGCCTGCTTT		
qRT-ABF3F1	CGCTCGCAAGCAAGCTTATAC	*AtABF3*	AT4G34000
qRT-ABF3R1	CAGAGGCTCCAGAAGCTGATTT		
qRT-ABF4F1	GCAGAAGAATGAGCAGCTGAAAG	*AtABF4*	AT3G19290
qRT-ABF4R1	GGAGTCTGGTTCATCCGTTCTT		
qRT-AtActin2F1	CGCTGACCGTATGAGCAAAG	*AtACT2*	AT3G18780
qRT-AtActin2R1	CCTTGGAGATCCACATCTGCT		
qRT-AtMON1F1	AAGGATTGGGACCCCACAAG	*AtMON1*	AT2G28390
qRT-AtMON1R1	TTATCGCCATCGCCTTGTCT		

Supplementary Table S2. Primers used for *AhAL* gene cloning.

Primer name	Sequence (5→3')
AhALF1	GYATHAAYTTYGCYAGRGATGG
AhALR1	GCWGGDGTDATYTTMACACA
SMART II Oligo	AAGCAGTGGTATCAACGCAGAGTACGCGGG
3'-CDS	AAGCAGTGGTATCAACGCAGAGTAC(T)30V N
5'-CDS	(T)25V N
UPML	CTAATACGACTCACTATAGGGCAAGCAGTGGTATCAACGCAGAGT
UPMS	CTAATACGACTCACTATAGGGC
NUP	AAGCAGTGGTATCAACGCAGAGT
SF1	TAAGGACAAGCCTAGTGTGGATAGTG
NSF1	GGCAAGTGATGGGCAGATCAAGAG
SR1	CTCTTGATCTGCCCATCACTTGCC
NSR1	CTATCCACACTAGGCTTGTCCTTAAC
SF2	ACAATTGAAAGAGAGATCTTCGACTCC
NSF2	CAAATCGGGCGGCAACAAGTACTC
SR2	TGAGTACTTGTTGCCGCCCGATTTG
NSR2	TGATTAGGAGTCGAAGATCTCTCTTTC
SF3	AGCAATCAAATTCAAAAGAGAAGACATC
NSF3	CCAAATATTCTTCAAAAGGGCCACAAC
SR3	GTTGTGGCCCTTTTGAAGAATATTTGG
NSR3	TGATGTCTTCTCTTTTGAATTTGATTGC
SF4	CCATCACGACAGCCTGAGAACCAC
NSF4	AGAGTGGTGGGGAGGAAGATGAAG
SR4	GTGGTTCTCAGGCTGTCGTGATGG
NSR4	GAGGACCAGAATGATCCTTTGGTTG

Supplementary Table S3. Core cis-elements (GTGGNG/GNGGTG) in the promoter regions of AhAL1-downregulated genes.

Gene name	Locations of cis-element
ABI1	GTGGCG (-1746 bp, -), GAGGTG (-2084 bp, +; -2711 bp, +)
ABI2	GGGGTG (-699 bp, +), GTGGCG (-1410 bp, +; -2051 bp, -), GAGGTG (-1535 bp, +), GTGGTG (-2384 bp, +), GCGGTG (-2389 bp, +)
AHG3	GTGGGG (-838 bp, +), GTGGTG (-1884 bp, +; -2922 bp, +)
HAB1	GTGGTG (-32 bp, +; -37 bp, +), GAGGTG (-2030 bp, +), GTGGAG (-2853 bp, +)
HAB2	GTGGAG (-622 bp, +; -1407 bp, +), GTGGTG (-1079 bp, +), GCGGTG (-1354 bp, +), GAGGTG (-1410 bp, +), GTGGTG (-2537 bp, +)
GRF7	GTGGGG (-388 bp, -), GAGGTG (-1457 bp, -), GCGGTG (-1488 bp, +)
DREB1C	GTGGGG (-214 bp, -), GGGGTG (-944 bp, -), GTGGCG (-1257 bp, +), GTGGAG (-1581 bp, +; -2455 bp, +), GCGGTG (-2853 bp, +)

Supplementary Table S4. Probes used for EMSA assay of AhAL1 DNA-binding activities.

Gene name	Probe sequence (5→3’)
ABI1	ATGAACCTTTTTTTCTTTCGATCAAATCTCTCCCGCCACCCAACTAACAC
ABI2	CAAAACTTCTATAAAGATACCCAATAATAATGGAGGGGTGACACGATAGT
AHG3	TATTACTAGATTTTTCTTATATGTTTTAAGGGTAGTGGGGCTGACCTATC
HAB1	AGTCCATCTTTGAAATTTAAAGCTTTGTTGTGGTGTGGTGTGGAAATCTC
HAB2	GGTCGTTGTCACATAAAGAAACTTGGGATTCCGTGGAGTGCTTATAATTT
GRF7	CATCAATCCACAAACTAGATTCATAGAAAACATCCCCCACCGTTAGATCG
DREB1C	CGTGGCATTCACAGAGACAGAAACTCCGCGTTCGACCCCACAAATATCCA

The core cis-elements in the promoter regions for AhAL1 binding are underlined.

Supplementary Table S5. Primers used for gene cloning and vector construnction.

Primer name	Sequence (5→3')	Restriction sites
AhAL1F1	GGATCCATGTCATCCTCAAATCCTCGAAC	*Bam*H I
AhAL1R1	GGTACCGTCGACTCAGTGACTACGCCCTGTC	*Kpn* I + *Sal* I
AhAL1R2	GTCGACGTGACTACGCCCTGTCTTC	*Sal* I
AhAL2F1	GGATCCATGGATATTGGAGCGCAGTAC	*Bam*H I
AhAL2R1	GAGCTCGTCGACTCATGGCCGAGCCCTCTTG	*Sac* I + *Sal* I
AhAL2R2	GTCGACTGGCCGAGCTCTCTTGTTAC	*Sal* I
AhAL3F1	GGATCCATGGACGGAAATACGCAGTAC	*Bam*H I
AhAL3R1	GGTACCGTCGACTCAAGGCCTTGCTCTTTTGTTG	*Kpn* I + *Sal* I
AhAL3R2	GTCGACAGGCCTTGCTCTTTTGTTGCTG	*Sal* I
AhAL4F1	GGATCCATGGAAGGATTGCCGCAGAAC	*Bam*H I
AhAL4R1	GGTACCGTCGACTCAAACACGAGCTCTCTTGC	*Kpn* I + *Sal* I
AhAL4R2	GTCGACAACACGAGCTCTCTTGCTAC	*Sal* I
ActinF1	ATGGTNAARGCNGGNTTYGC	
ActinR1	TCNGGNGGNGCNACNACYTT	
